# A high-throughput screening approach to discover potential colorectal cancer chemotherapeutics: repurposing drugs to identify novel disruptors of 14-3-3 proteins

**DOI:** 10.1038/s41419-025-08150-6

**Published:** 2025-11-10

**Authors:** Siyi He, Daniel Meister, Samra Khan, Azam Mohammadzadeh, Luis Delgadillo Silva, Guy A. Rutter, John F. Trant, Gareth E. Lim

**Affiliations:** 1 Department of Medicine, Faculty of Medicine, Université de Montréal, Montréal, QC Canada; 2 Cardiometabolic axis, Centre de Recherche du Centre hospitalier de l’Université de Montréal (CRCHUM), Montréal, Québec Canada; 3 Department of Chemistry and Biochemistry, University of Windsor, Windsor, ON Canada; 4 Department of Diabetes, Endocrinology and Medicine, Faculty of Medicine, Imperial College, London, UK; 5 LKC School of Medicine, Nanyang Technological College, Singapore, Republic of Singapore; 6 Research Institute of the McGill University Health Centre, Montreal, Canada; 7 WE-Spark Health Institute, Windsor, ON Canada; 8 Binary Star Research Services, LaSalle, ON Canada; 9 Department of Biomedical Sciences, University of Windsor, Windsor, ON Canada

**Keywords:** High-throughput screening, Cancer

## Abstract

Selectively inducing apoptosis of cancer cells is an effective therapeutic strategy, but the success of existing chemotherapeutics is compromised by emergent tumor cell resistance and systemic off-target effects. Therefore, the discovery of new pro-apoptotic compounds with minimal systemic side effects remains an urgent need. 14-3-3 proteins are molecular scaffolds that serve as important regulators of cell survival. We previously demonstrated that 14-3-3ζ can sequester BAD, a pro-apoptotic member of the BCL-2 protein family, in the cytoplasm to inhibit the induction of apoptosis. Despite 14-3-3ζ being a critical regulator of cell survival, the identification of molecules that potently disrupt 14-3-3ζ actions has yet to materialize as a chemotherapeutic approach. Herein, we established a BRET-based, high-throughput drug screening approach (Z’-score = 0.52) to identify molecules that disrupt the binding of 14-3-3ζ to a BAD-derived fragment containing serine residues critical for their interactions. A drug library containing 1971 compounds was used for screening, and the capacity of identified hits to induce cell death was examined in NIH-3T3 fibroblasts and colorectal cancer cell lines, HT-29 and Caco-2. These results were mechanistically supported by both in silico structural analysis that suggest the possible mode of binding and direct biophysical measurements that demonstrate concentration-dependent target engagement. Terfenadine, penfluridol, and lomitapide have potential to either be repurposed as chemotherapeutics, or more likely, used as starting points for novel lead development. The described assay cascade demonstrates the feasibility of both expanding on these compounds and identifying novel disruptors of 14-3-3ζ to develop pro-apoptotic agents to treat pathogenic aberrant cell growth.

## Introduction

Apoptosis, or programmed cell death, is a highly regulated process of cell suicide. During apoptosis, cells break down into apoptotic bodies and are eventually engulfed by phagocytes like macrophages and neutrophils [1]. With limited leakage of a cell’s content into the extracellular environment, apoptosis can minimize the damage to surrounding cells. The identification of molecules that safely and selectively induce apoptosis holds significant potential in treating a variety of conditions, such as cancer, infectious diseases, and autoimmune disorders [2–4].

In Canada, colorectal cancer (CRC) ranks as the second most deadly cancer among men and third among women [5]. It is estimated that in 2025 in the United States of America, there will be 154,270 newly diagnosed cases of CRC and 52,900 deaths from CRC [6]. CRC carcinogenesis originates from either the colon or the rectum, and most malignant adenomas develop from benign polyps [7]. Current therapies for CRC involve chemotherapy, radiation therapy, and surgery; however, resistance to existing chemotherapeutics often leads to poor clinical outcomes [8]. Accordingly, it remains important to discover new mechanisms and compounds that can selectively induce apoptosis in CRC cells.

An example of a chemotherapeutic that exploits the intrinsic pathway of apoptosis is Venetoclax (ABT-199), a specific inhibitor of the anti-apoptotic protein, BCL-2 [9]. The effectiveness of ABT-199 in treating chronic lymphocytic leukemia and acute myeloid leukemia highlights the potential of targeting the actions of members of the BCL-2 protein family to treat cancers. We previously demonstrated that 14-3-3ζ, a member of the 14-3-3 scaffold protein family, plays an essential role in maintaining the survival of murine MIN6 insulinoma cells through its inhibitory actions on pro-apoptotic BCL-2 proteins, such as BAD [10]. In healthy cells, 14-3-3 proteins sequester BAD in the cytoplasm by interacting with phosphorylated Ser112 and Ser136 residues on BAD [11]. However, the induction of cell death or prolonged cell stress often results in these serine residues becoming dephosphorylated, leading to the dissociation of 14-3-3 protein:BAD complexes. This allows BAD to translocate to the outer mitochondrial membrane, where it interacts with other BCL-2 proteins, mainly through displacing pro-apoptotic BAX and BAK from anti-apoptotic BCL-2 and BCL-xL, to initiate apoptosis [12–14].

14-3-3 proteins have been reported to have important roles in cancer cell survival [15]. Alterations in 14-3-3 protein expression, especially the 14-3-3ζ isoform, have been observed in a variety of cancers, such as those of colon, breast, lung, and pancreas [16]. Overexpression of 14-3-3ζ may mediate tumor resistance to chemotherapy due to its anti-apoptotic functions [17]. Depletion of 14-3-3ζ has been found to induce the apoptosis of CRC cells in vitro and in vivo [18], suggesting that identifying or developing novel inhbitors of 14-3-3ζ that disrupt interactions with BAD may represent a promising approach towards the treatment of CRC and other cancers.

Drug development involving de novo synthesis and validation of novel chemical entities is an incredibly expensive and time-consuming endeavor with a low success rate [19–21]. From preclinical studies to clinical trials, and ultimately to approval by the US Food and Drug Administration (FDA), the estimated average cost per drug was over $1.5 billion between 2009 and 2018, with a development time of up to over 20 years [19, 20]. Much of this cost and time is driven by failure, as only 10% of drugs that enter phase I clinical trials are approved [21]. Given the challenges of new drug development, repurposing already approved compounds for new indications is an attractive strategy, to save both time and costs [22–24]. The drugs themselves can be repurposed, or more likely, they offer excellent physiologically-tolerated scaffolds as great starting points for lead development [25, 26]. To date, the most comprehensive compound screen aimed at identifying disruptors of 14-3-3 protein:BAD protein-protein interactions (PPIs) was performed with a time-resolved fluorescence resonance energy transfer (TR-FRET)-based approach. Although 16 hits were discovered from over 52,100 examined compounds, an important caveat was that this assay was based on a cell-free system involving recombinant proteins [27]. It was not possible to discern if identified hits would act via receptor-mediated pathways or be transported into a cell to directly disrupt 14-3-3 protein:BAD PPIs. Moreover, essential follow-up assays to evaluate these activities were not conducted.

Herein, we have developed an innovative BRET (bioluminescence resonance energy transfer)-based biosensor to detect 14-3-3 protein:BAD PPIs in intact, living cells [28]. Using this sensor and a drug library containing 1,971 compounds that were either FDA-approved or received orphan drug designation, we first identified 101 hits through a high-throughput screening (HTS) approach in NIH-3T3 fibroblasts. We also examined the possible binding modes of the top compounds using molecular docking simulations and compared them to known inhibitors, in order to better understand the molecular contexts of our constructs. We next evaluated the capacity of these hits to induce cell death, and 41 compounds emerged as potential candidates. Based on their original indications and routes of administration, we selected 13 of these drugs for further assessment of their effectiveness in inducing apoptotic cell death in the well-characterized HT-29 and Caco-2 CRC cell lines. Top candidates identified by the combination of the in silico and in vitro methods were then confirmed to be directly interacting with 14-3-3ζ using surface plasmon resonance. Our screening workflow has identified terfenadine, a withdrawn antihistamine, penfluridol, a 1^st^ generation antipsychotic, and lomitapide, a non-statin cholesterol control medication, as candidate molecules that can be potentially repurposed as chemotherapies for treating malignant cell growth.

## Materials and methods

### BRET sensor construction

The original plasmids containing 14-3-3ζ, BAD, and BAD mutants were kind gifts from Dr. Herman Spaink and Dr. Aviva M Tolkovsky, respectively [29, 30]. To conjugate mTurquoise, a cyan fluorescent protein (CFP) to the C- or N-termini of 14-3-3ζ, 14-3-3ζ was subcloned into pmTurquoise2-N1 (Addgene, Massachusetts, USA; plasmid #54843) using restriction enzymes EcoRI (NEB, Massachusetts, USA; #R0101S) and AgeI (NEB; #R0552S), and also into pmTurquoise2-C1 (Addgene; plasmid #60560) using EcoRI and KpnI-HF (NEB; #R0145S), respectively. pmTurquoise2-N1 and pmTurquoise2-C1 were gifts from Michael Davidson and Dorus Gadella [31]. For the conjugation of Renilla luciferase-8 (Rluc8) to the C-terminus of 14-3-3ζ, Rluc8 was subcloned from pcDNA-Rluc8 (a kind gift from Dr. Jace Jones-Tabah and Dr. Terry Hébert, McGill University) and used to replace the mTurquoise of constructed 14-3-3ζ-mTurquoise using AgeI and NotI-HF (NEB; #R3189S). To conjugate Rluc8 to the N-termini of 14-3-3ζ, Rluc8 was subcloned to the original 14-3-3ζ-containing plasmid using EcoRI and KpnI-HF [29]. Specific primers were used to generate truncated forms of BAD, which were then subcloned into pmCitrine-C1 (Addgene; plasmid #54587) and pmCitrine-N1 (Addgene; plasmid #54594) using EcoRI and BamHI (NEB; #R0136S). This process attached mCitrine, a yellow fluorescent protein (YFP), to the N- and C-termini of BAD, respectively. pmCitrine-C1 and pmCitrine-N1 were gifts from Robert Campbell, Michael Davidson, Oliver Griesbeck, and Roger Tsien [32]. To construct bi-directional plasmids, Rluc8-conjugated 14-3-3ζ and BAD variants conjugated to mCitrine were subcloned to the multiple cloning site 2 (MCS-2) of the pBI-CMV1 vector (Takara, Shiga, Japan; #631630), using EcoRI and XbaI (NEB; #R0145S) and to its MCS-1 using MluI-HF (NEB; #R3198S) and SalI-HF (NEB; #R3138S), respectively. Primers were designed with SnapGene Viewer (7.1.0) and synthesized by Integrated DNA Technologies (IDT, California, USA). Phusion® High-Fidelity DNA Polymerase (NEB; #M0530S) was used for PCR amplification. QIAquick Gel Extraction Kit (Qiagen, Hilden, Germany; #28704) was used to recover DNA products from agarose gels. T4 DNA ligase (NEB; #M0202S) was used to insert genes into vectors. Primer sequences are listed in Table 1.

**Table 1:** Primers and restriction enzymes for the construction of BRET sensor.

Gene	Primer
5’-14-3-3ζ	ATGGATAAAAATGAGC
3’-14-3-3ζ	ATTTTCCCCTCCTTCTCCTG
5’-BAD	ATGGGAACCCCAAAGCAG
3’-BAD	TGGATCCTGGGAGGGGGTG
3’-BAD-136F	CGCTGCCCAGAGATTGGG
5’-112-136F	ATGGAGACTCGGAGTCGC
5’-Rluc8	ATGGCTTCCAAGGTGTACGAC
3’-Rluc8	CTGCTCGTTCTTCAGCACGC
5’mCitrine	ATGGTGAGCAAGGGCGAG
3’-mCitrine	CTTGTACAGCTCGTCC
5’-mTurquoise	ATGGTGAGCAAGGGCG
3’-mTurquoise	CTTGTACAGCTCGTCCATGCC

### Cell culture

NIH-3T3 cells were kindly provided by Dr. Marc Prentki (CRCHUM, Montreal, Canada) and maintained in 25 mM glucose DMEM (Gibco, Massachusetts, USA; #11995065) supplemented with 10%FBS (Gibco; #12483020) and 1% penicillin-streptomycin (Gibco; #15140122). HT-29 and Caco-2 cells were kind gifts from Dr. Petronela Ancuta (CRCHUM, Montreal, Canada) and maintained in Advanced MEM (Gibco; #12492013) supplemented with 20% FBS and 1% penicillin-streptomycin or McCoy 5A media (Gibco; #16600082) supplemented with 10%FBS and 1% penicillin-streptomycin, respectively. All cells were cultured in a humidified incubator at 37 °C with 5% CO_2_ and passaged upon reaching 70–80% confluency, and they were previously tested and deemed to be free of mycoplasma contamination. All studies were repeated with cells at different passages to ensure reproducibility.

### Plasmid and siRNA transfection

Plasmid DNA was amplified in Subcloning Efficiency DH5α competent cells (Invitrogen, Massachusetts, USA; #18265-017) and extracted using QIAprep Spin Miniprep Kit (Qiagen; #27104) or PureLink™ HiPure Plasmid Maxiprep Kit (Invitrogen; #K210007), following the manufacturer’s protocol. The purity and concentration of each plasmid were assessed with a NanoDrop™ Lite Spectrophotometer (Invitrogen). Two days before transfection, cells were plated in 12-well plates with a density of 30,000 cells/well or proportionally adjusted based on the surface area of different well sizes. Plasmids were transfected with Lipofectamine™ 3000 Transfection Reagent (Invitrogen; #L3000015), according to the manufacturer’s instructions.

To deplete BAD, HT-29 and Caco-2 cells were seeded in 12-well plates at a density of 150,000 cells per well. After 24 h, siRNAs targeting *BAD*, si1862 (si*BAD*-1) and si1863 (si*BAD*-2) (Thermo Fisher Scientific; #4390824), were transfected into cells using the RNAiMAX transfection reagent (Invitrogen; #13778075) according to the manufacturer’s protocol.

### Co-immunoprecipitation (Co-IP) and immunoblotting

NIH-3T3 cells were plated in 10 cm culture dishes at a density of 550,000 cells per dish. Transfection was conducted 48 h post-seeding. At 48 h post-transfection, DMSO or FTY-720 (20 μM) was added to the culture media and incubated with cells expressing the BRET-sensor for 3 h. Cell lysates were collected using RIPA buffer (0.9% NaCl, 1% v/v triton X-100, 0.5% sodium deoxycholate, 0.1% SDS, and 0.6% tris base), supplemented with protease inhibitor cocktail (Sigma-Aldrich; #P8340). The protein concentration was determined using the Bradford assay. Equal amounts of protein (100 μg) were subjected to immunoprecipitation using 1 μg anti-GFP antibody per sample (Invitrogen; #A-11120) and protein A/G magnetic beads (Thermo Fisher Scientific, Massachusetts, USA; #80104G), according to the manufacturer’s instructions. Antibodies against GFP were used to precipitate mCitrine due to the high degree of sequence homology. Immunoprecipitated proteins were eluted by boiling the beads in 1 X Laemmli protein sample buffer (Bio-Rad, California, USA; #1610747) for 5 min. Eluted samples were resolved in 10% SDS-PAGE gels and transferred to PVDF membranes (Bio-Rad, California, USA; #10026933) using the Trans-Blot Turbo transfer system (Bio-Rad). Membranes were probed with anti-GFP (1:1000; Abcam, Cambridge, UK; #290) and anti-Renilla luciferase (1:1000; Abcam; #ab187338) antibodies. To assess depletion of BAD, cell lysates were collected 48 h following transfection with siRNA, and equal amounts (40 μg) of protein were resolved on 12% SDS-PAGE gels and transferred to PVDF membranes using the Trans-Blot Turbo transfer system. BAD and β-actin were probed using anti-BAD (1:1000; Cell Signaling Technology, Massachusetts, USA; #9292) and anti-β-actin (1:2000; Cell Signaling Technology, #3700) antibodies. Following incubation with HRP-conjugated secondary antibodies (Cell Signaling Technology, Massachusetts, USA; Anti-mouse IgG, #7076; Anti-Rabbit IgG, #7074). Protein bands were detected using Clarity Max western ECL substrate. Membranes were visualized with the iBright 1500CL image system. Densitometry was performed with ImageJ2 (Ver 2.14.0).

### Confocal imaging and colocalization analysis

Confocal images were acquired with a Leica TCS-SP5 inverted microscope using an HCX PL APO CS 100×/1.4 oil objective with the Las-AF software. Excitation was performed using the 458 nm and 514 nm laser lines of an Argon laser for CFP and YFP, respectively, and a 633 nm HeNe laser for TOPRO-3. Detection bandwidth was set to 468–500 nm for CFP using a HyD detector under the standard mode, 524–623 nm for YFP using a PMT, and 643-748 nm for TOPRO-3 using a HyD detector under the standard mode. A sequential acquisition consisting of CFP and YFP in sequence 1, and TOPRO-3 in sequence 2 was performed. A line average 4 was applied for each sequence. Images were acquired at zoom 2.5 with a 400 Hz scan speed and the final images are 12 bits, 1024 × 1024 pixels (axial pixel size of 60 nm).

To visualize BAD translocation, 35 mm glass-bottom dishes (ibidi, Gräfelfing, Germany; #81158) were coated with 0.1 mg/mL Poly-d-lysine hydrobromide for 2 h at 37 °C. After coating, dishes were washed twice with sterilized water. Subsequently, cells transfected with BAD-mCitrine were seeded at a density of 20,000 cells per dish and incubated for 24 h to allow for cell attachment. For live-cell imaging, cells were then incubated for 1 h with the mitochondrial membrane potential dye tetramethylrhodamine ethyl ester (TMRE, Invitrogen; #T668) at a final concentration of 100 nM, according to the manufacturer’s instructions. Images were acquired on a Zeiss (Oberkochen, Germany) LSM-900 Airyscan inverted confocal microscope equipped with an environmental incubation system, laser lines including 488 and 561 nm, and an oil immersion 60×/1.40 Plan achromat objective. The signal from the YFP fluorophore (ex. 488 nm, det. 525–542 nm) and the TMRE (ex. 561 nm, det. 600–700 nm) were collected in super-resolution mode. The cell viability dye, DRAQ (BioLegend, California, USA; #424101), was added to the medium at a 1:1000 dilution from the commercial stock and incubated at room temperature for 30 min prior to live-cell imaging. To quantify the colocalization of BAD-mCitrine and the mitochondria labeled with the TMRE, the JACoP plugin in ImageJ was employed [33]. A correlation analysis was performed based on Pearson’s coefficient between pixel-gray values for the two channels.

### Protocol for the high-throughput screening (HTS) of drugs

The Z’-score, which evaluates the robustness of an HTS assay readout, was calculated according to standard protocols [34]. For the HTS, 384-well plates were coated with 100 μg/mL poly-l-ornithine (Sigma, Missouri, United States; #P3655) overnight at 37 °C, followed by rinsing with sterilized water. NIH-3T3 cells were transfected with the BRET sensor two days prior to experimentation, followed by replating onto the pre-coated 384-well plates at a density of 20,000 cells/well. After 24 h of incubation at 37 °C, growth media was replaced by Krebs buffer (pH 7.4). A robotic liquid handling system (Eppendorf, Hamburg, Germany; epMotion 5075) was used for appropriately diluting and adding the drug library (APExBIO, Texas, USA; #L1021, 2021 edition) to corresponding wells. Cells were incubated (37 °C) for 3 h with each drug at final concentrations of 200 μM, 20 μM, 2 μM, and 200 nM, followed by the addition of coelenterazine-h (NanoLight Technology, Arizona, USA; #301), the substrate of Rluc8, to each well (final concentration of 5 μM). After incubation for 10 min, emissions from Rluc8 at 460 nm and mCitrine at 535 nm were measured (Perkin-Elmer, Massachusetts, USA; 1420 Multilabel counter). BRET ratios were calculated by the 535/460 nm ratio of emission readings [35], and BRET reduction was quantified by normalizing the BRET ratio of drug-treated cells BRET_drug_ to the BRET ratio of solvent-treated control cells BRET_control_, using the formula: (BRET_control_ – BRET_drug_) / BRET_control_.

### Cell death and apoptosis assays

Cells were plated at a density of 20,000 cells/well into 96-well plates (PerkinElmer; #6055302) and incubated overnight at 37 °C before the addition of the drugs. One hour prior to imaging, Hoechst 33342 (Invitrogen, #H3570) and propidium iodide (Invitrogen; #P3566) were added to the wells to achieve final concentrations of 50 ng/ml and 0.5 μg/ml, respectively. To measure apoptosis, AlexaFluor 488-conjugated Annexin V (ThermoFisher, Massachusetts, USA; #A13201) was also added at a dilution of 1:400 from the commercial stock. A high-content imaging system (PerkinElmer; Operetta CLS), was used to quantify cell death and apoptosis by calculating the number of propidium iodide-positive cells relative to Hoechst-positive cells, and the number of Annexin V-positive cells relative to Hoechst-positive cells, respectively. To assess caspase-dependent apoptosis, cells were pre-treated for 30 min with the caspase inhibitor Z-VAD-FMK (BD Biosciences, New Jersey, USA; #550377; 20 μM) or the negative control Z-FA-FMK (BD Biosciences; #550411; 20 μM) prior to the incubation of drugs.

### Computational methodology

#### Structure preparation

Structures of 14-3-3ζ (PDB Code *2C1J*, chosen as it had no missing components or loops), mCitrine (PDB Code *3DQO*), and Rluc8 (PDB Code *7OMO*) were obtained from the protein data bank while full-length BAD was calculated using AlphaFold2 under standard parameters (Deepmind, London, UK) [36–38]. BAD is a poorly structured protein and a full-length crystal structure is unavailable. Solvent and, if present, ligands, were removed from available structures that were prepared using the protein preparation module in Schrödinger (New York, NY). The protonation states of proteins were set, assuming a pH of 7.4, and the structures were then minimized. As the confidence of the structure generated by Alphafold was low, full-length BAD was subjected to a 500 ns Gaussian accelerated molecular dynamics (GaMD) simulation to ensure an equilibrated structure. The BAD 112-136F fragment spanned M104-A144 and was similarly obtained using Alphafold and prepared using Schrodinger. As the fragment is smaller and more flexible, three 500 ns GaMD simulations were performed to ensure proper sampling of conformations. GaMD simulations have been shown to significantly enhance sampling and have been used to accurately fold peptides [39]. To generate the 14-3-3ζ-RLuc and BAD-mCitrine constructs, the missing N terminal sequence of 14-3-3ζ was built using Schrödinger and connected to Rluc8 using the Protein Linker Design module. Similarly, full-length BAD and truncated BAD were attached to mCitrine.

#### Gaussian accelerated molecular dynamics

Molecular dynamic (MD) simulations were performed using Amber20 [40]. Structures were prepared using Leap with the ff19SB forcefield for the proteins and the OPC water model [41]. The protein was surrounded in a 12 Å-*per* side octahedral periodic solvent box, and charges were neutralized using either Na^+^ or Cl^-^ ions as appropriate. Na^+^ and Cl^-^ were then added to achieve a 0.15 M ionic strength concentration for the water box. Three minimization steps, each with 10,000 steps conjugate gradient & 10,000 steps steepest descent with decreasing restraints on the protein, of 100, 3, and 0 kcal·mol^−1^·Å^−2^ l were performed. For the remaining steps, SHAKE was used to constrain hydrogen bonds, along with a nonbonded cutoff of 12 Å, with a 2 fs time step [42]. The pressure was regulated using the Berendsen barostat, and the Langevin thermostat with a collision frequency of 2 ps^−1^ was used for temperature. The system was then heated to 300 K over 50 ps with a harmonic restraint of 1 kcal·mol^−1^·Å^−2^ on the protein. This was followed by two density equilibration steps, 2 fs time steps, to allow for adjustment of periodic box conditions. The first was for 50 ps with a restraint of 2 cal·mol^−1^·Å^−2^ on the protein and the second was for 200 ps with no restraint. For the GaMD simulation, a dual boost on both dihedral and total potential energy was used with the threshold energy set to the lower bound. For equilibration, 200,000 steps of conventional MD with a 2 fs time step were performed to equilibrate the system, followed by 1,000,000 steps of initial conventional MD simulations to collect potential energies. 800,000 steps of preparation biasing MD steps were performed followed by 50 ns of GaMD equilibration. This was followed by 450 ns of GaMD simulations. First and second potential boosts of 3 kcal·mol^−1^ were utilized for all GaMD simulations.

#### Clustering

Trajectories were clustered using cpptraj and the kmeans clustering algorithm based on the backbone atoms of the protein. For full-length BAD, the clusters were similar with minor differences in the loop containing residues Ser112 and Ser136 and the orientation of the residues [43]. The lowest energy top pose was chosen for use in further studies as Ser112 and Ser136 both point away from the protein core and are solvent exposed, while in the other clusters, the side chains are rotated inwards towards BAD and cannot form interactions with 14-3-3ζ. For truncated BAD, due to the flexible nature of the protein all of the top 5 poses were used for further study.

#### Docking protocol

Structures of small molecule inhibitors were prepared using the ligprep module in Schrödinger at a pH of 7.4. If multiple protonation states were possible, all were used in subsequent docking. Induced fit docking was performed targeting the groove of 14-3-3ζ using the standard protocol in Schrödinger with residues within 5 Å of ligand poses being refined with XP precision for Glide redocking.

#### Protein–protein docking

Phosphorylated full-length BAD or truncated BAD was docked to the 14-3-3ζ dimer using the Piper protein–protein docking module in Schrödinger. No constraints were used for full-length BAD, while constraints on the BAD fragment were used to ensure that the BAD fragment docked to 14-3-3ζ and not the conjugated mCitrine.

#### Molecular dynamics simulations of 14-3-3ζ-BAD complexes

To observe the distances and interactions between mCit and Rluc8, MD simulations were performed. Due to the large size of the systems, explicit solvation could not be used due to runtime constraints. Instead, implicit solvation was used. Ff14SB was used for the protein with the OBC-2 solvent model with a salt concentration of 0.15 M, no nonbonded cutoff, and a 2 fs time step. Similar minimization and heating steps were performed as above and similar parameters were used for equilibration, however, periodic boundary conditions were not used.

### Biophysical characterization of 14-3-3ζ interactions with lead candidates

#### Expression and purification of 14-3-3ζ

The published protocol of Higuchi & Ottmann as followed with modifications to express 14-3-3ζ [44]. *Escherichia coli* BL21 LOBSTR-RIL cells were transformed with a plasmid encoding His-14-3-3ζ. The transformed cells were grown in TB medium containing appropriate antibiotic at 37 °C with shaking at 200 rpm until the culture reached an optical density of 0.6–0.8 at 600 nm (OD600). Protein expression was induced by the addition of isopropyl β-d-1-thiogalactopyranoside to a final concentration of 0.4 mM. Subsequently, the temperature was decreased to 16 °C and the culture incubated for another 16 h. Cells were harvested by centrifugation at 7000 × *g* for 20 min at 21 °C. The supernatant was discarded, and the cell pellet containing 14-3-3ζ was mixed and resuspended in lysis buffer (50 mM Tris, pH 7.80), 500 mM NaCl, 5 mM imidazole, 5% glycerol, 1 mM PMSF & 1 mM Benzamidine. Cells were lysed by sonication on ice using a probe sonicator (20 cycles of 10 s on, 20 s off, amplitude 70%). The lysate was centrifuged at 30,000 × *g* for 30 min at 4 °C to remove cell debris. The supernatant was collected for further purification. The 14-3-3ζ was isolated from the lysate by affinity chromatography (Ni-NTA beads, Qiagen). For further purification, the eluant was subjected to size exclusion chromatography using a Superdex 75 gel filtration column (HiLoad 16/600, GE Healthcare), pre-equilibrated with SEC buffer (20 mM Tris, pH 7.5, 150 mM NaCl, 1 mM TCEP). The protein solution was concentrated by ultrafiltration, flash frozen, and stored at −80 °C.

#### Surface plasmon resonance (SPR) assay

The general protocol for evaluating 14-3-3ζ was adapted from Hartman et al*.* and modified for our instrument and sensors [45]. The SPR experiments were performed using a Open surface plasmon resonance instrument (OpenSPR^TM^, Nicoya, Kitchener-Waterloo, Canada), and high sensitivity-nitrilotriacetic acid (HS-NTA) sensors. The sensor chip surface was first activated with 40 mM of NiCl_2_ at a flow rate of 20 μL/min using 20 mM HEPES pH 7.8, 150 mM NaCl and 1% DMSO as the running buffer. All running buffers were filtered and degassed prior to use. 100 μg/mL His-14-3-3ζ protein in 20 mM HEPES pH 7.8, 150 mM NaCl and 1% DMSO was injected at 8 μL/min flow rate for 800 s contact time and immobilized until the instrument registered 2900–3500 RU (activation and protein immobalization spectrograms are provided as Supplementary Fig. 10). Drugs were dissolved in DMSO and were diluted with 20 mM HEPES pH 7.8, 150 mM NaCl (final DMSO concentration of 1% v/v) and were injected at a flow rate of 50 μL/min. Single-cycle kinetics were applied for KD evaluation and binding affinity using TraceDrawer v.1.8.1 (Ridgeview Instruments AB).

### Synthetic organic chemistry

#### General methods and materials for the synthetic chemistry

Solvents were purchased from Caledon Labs (Caledon, Ontario), Sigma-Aldrich (Oakville, Ontario) or VWR Canada (Mississauga, Ontario). Other chemicals were purchased from Sigma-Aldrich, AaronChem, AK Scientific, Oakwood Chemicals, Alfa Aesar or Acros Chemicals and were used without further purification unless otherwise noted. Anhydrous DMSO was purchased from Millipore Sigma. Anhydrous THF was obtained by distillation over benzophenone-sodium. All heated reactions were conducted using oil baths on IKA RET Basic stir plates equipped with a P1000 temperature probe. Thin layer chromatography was performed using EMD aluminum-backed silica 60 F254-coated plates and were visualized using either UV-light (254 nm). Column chromatography was carried out using standard flash technique with silica (Siliaflash-P60, 230–400 mesh Silicycle) under compressed air pressure. ^1^H NMR spectra were obtained at 500 MHz. NMR chemical shifts (δ) are reported in ppm and are calibrated against residual solvent signals of DMSO-d_6_ (δ 2.50), or methanol-d_4_ (δ 3.31). Spectra were processed using MNOVA 14.1.2 (Mestrelab Research S.L., Santiago de Compostela, Spain). Fourier transform was conducted using a linear phase shift group delay, an exponential-fit 1 Hz apodization, and a zero-filling linear prediction of 65,536 points. An automatic phase correction was applied and manually refined to provide a flatter baseline. A baseline correction, employing a Whittaker smoothing function with a 2.60 Hz filter and a smoothing factor of 16,384 was employed on the spectrum between −2.00 and 15.00 ppm. Integration was measured using the manual integration tool and referenced to a relevant aliphatic proton. Multiplet analysis was conducted using the algorithm as implemented in the software, confirmed by manual analysis. Specific experimental protocols for the synthesis of BV01 (Supplementary Scheme 1) and related characterization data (Supplementary Figs. 13, 14 and 15) are provided with the Supporting Information.

### Statistical analysis

R programming (4.3.2) was used for generating scatter density plots. Harmony® high-content analysis software (4.9) was used for processing data collected by the Operetta CLS system. Data were analyzed by GraphPad Prism (version 9.5.0), and the statistical significance of the results was examined by one-way ANOVA or two-way ANOVA with a Dunnett’s or a Tukey’s post hoc test. Statistical significance in the figures is shown as follows: *P < 0.05; **P < 0.01; ***P < 0.001; ****P < 0.0001. Data are presented as mean ± SEM.

## Results

### Inhibition of 14-3-3 proteins promotes BAD translocation

**Fig. 1:**
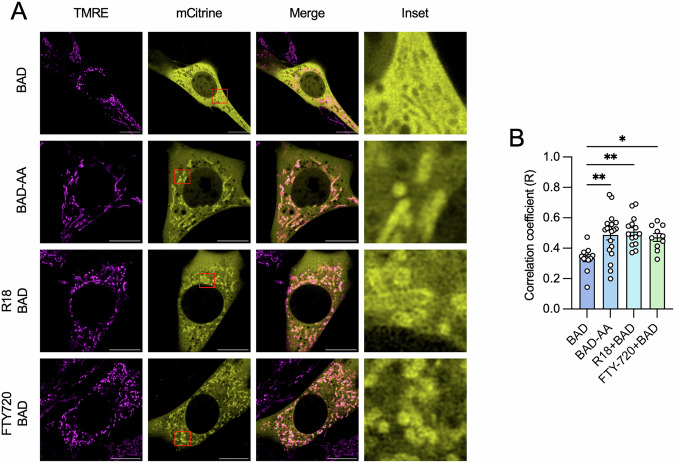
Disrupted 14-3-3 protein function promotes BAD translocation. **A** NIH-3T3 cells were made to express mCitrine-conjugated BAD (BAD) or BAD mutants harboring S112A and S136A double mutations (BAD-AA). BAD-expressing cells were treated with either R18 (10 µM) or FTY720 (2 µM) for 24 h. The mitochondrial membrane potential sensor TMRE (100 nM) was added to the medium 1 h prior to live-cell imaging. Representative images were selected from three independent experiments, with at least three images were captured per dish in each experiment (Scale bar = 10 µm). **B** The co-localization of BAD and mitochondria in 1 A was assessed by calculating Pearson’s correlation coefficient (R). Each data point corresponds to a single cell. Statistical significance was determined using one-way ANOVA with Tukey’s post hoc test. *P < 0.05; ***P < 0.001.

The canonical mechanism by which 14-3-3 proteins regulate cell survival is through the sequestration of pro-apoptotic BCL-2 proteins, such as BAD, in the cytoplasm [46]. To visualize how 14-3-3 protein inhibition impacts BAD localization and subsequent translocation to mitochondria, BAD-mCitrine was transiently expressed in NIH-3T3 cells, followed by incubation with TMRE (tetramethylrhodamine, ethyl ester) to label mitochondria. R18 and FTY720, which are established 14-3-3 protein inhibitors that act through distinct mechanisms of action [47, 48], were used to inhibit 14-3-3 proteins. BAD-mCitrine-expressing cells or control mCitrine cells were exposed to R18 (10 µM) and FTY720 (2 µM) for 24 h, and localization of mCitrine was visualized by confocal microscopy. A BAD mutant containing S112A and S136A double mutations (BAD-AA), which prevent 14-3-3 protein:BAD PPIs, was used as a positive control. In the absence of 14-3-3 protein inhibitors, BAD-mCitrine was distributed diffusely throughout the cytoplasm, and inhibition of 14-3-3 proteins with FTY720 and R18 promoted BAD translocation to TMRE-labeled mitochondria. This redistribution was also observed in cells expressing the BAD-AA-mCitrine mutant (Fig. 1A). An increased degree of co-locolization between the signal of mCitrine and TMRE was found in BAD-AA-expressing cells, and BAD-expressing cells treated with R18 and FTY-720, as determined by Pearson’s correlation coefficient (R) (Fig. 1B). These observations indicate the essential role of 14-3-3 proteins in regulating the cytoplasmic sequestration of BAD. Importantly, these findings also demonstrate that if mCitrine is conjugated to BAD, no detrimental effects on its ability to translocate to mitochondria would occur.

#### Development of a BRET-based living-cell sensor to detect interactions between 14-3-3ζ and BAD

**Fig. 2:**
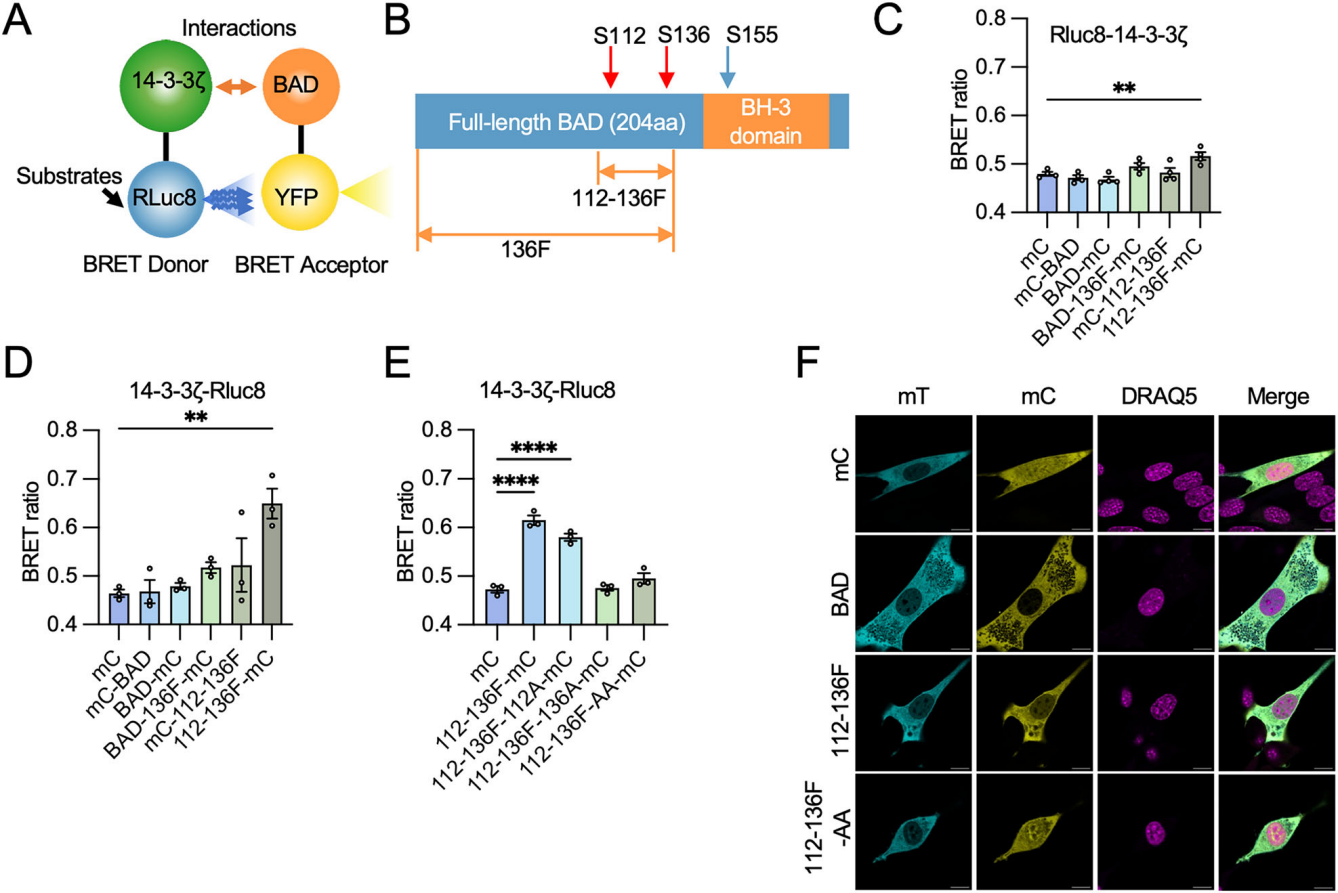
Construction of a BRET sensor to detect interactions between 14-3-3 and BAD. **A** Schematic of the BRET sensor: Rluc8 (BRET donor) and mCitrine (BRET acceptor) are conjugated to 14-3-3ζ and BAD or BAD variants, respectively. The interaction between 14-3-3ζ and BAD or its variants brings the donor and acceptor into close proximity, facilitating BRET. **B** Illustration of BAD and truncated forms of BAD. **C**, **D** Various pairings of BRET donor and acceptor were assessed. Rluc 8 was conjugated to either the N-termini (Rluc8-14-3-3ζ; C; *n* = 4 per group) or C-termini (14-3-3ζ-Rluc8; D; *n* = 3 per group) of 14-3-3ζ. mCitrine (mC) was linked to the N-termini of BAD (mC-BAD), the truncated BAD spanning Met106 to Ala144 (mC-112-136F), or to the C-termini of BAD (BAD-mC), truncated BAD extending from Met1 to Ala144 (BAD-136F-mC), and the fragment 112-136 F (112-136F-mC). **E** Single mutation S112A (112-136-F-112A) and S136A (112-136F-136A), and a double mutation combining S112A and S136A (112-136F-AA) were introduced to confirm the interactions between 14-3-3ζ-Rluc8 and 112-136F-mC (*n* = 3 per group). **F** In NIH-3T3 cells were co-transfected with 14-3-3ζ-mTurquoise (14-3-3ζ-mT) and either mC, BAD-mC, 112-136F-mC, or 112-136-F-AA-mC, and cell nuclei were visualized with 5 µM DRAQ5. Representative images were selected from three independent experiments, with three images were captured per dish in each experiment (Scale bar = 10 µm). Statistical significance was determined using one-way ANOVA with a Dunnett’s post hoc test, **P < 0.01,****P < 0.0001 when compared with mC (**C**–**E**).

To measure 14-3-3 protein:BAD PPIs in living cells, we focused our efforts on developing a BRET-based reporter whereby 14-3-3ζ and BAD would be conjugated to Rluc8 and mCitrine, respectively (Fig. 2A). As BRET is highly dependent on the close proximity of the donor (Rluc8) and acceptor (mC)itrine, we examined six BAD-mCitrine constructs whereby mCitrine was conjugated to either the N- or C-termini of full-length murine BAD, or of two truncated versions of BAD (Fig. 2B, Supplementary Fig. 1). The two truncated BAD fragments were BAD-136F, which comprises the N-terminus of BAD to residue A144, and BAD-112-136F which spanned from M104 to A144. We also ligated Rluc8 to both the N- and C-termini of 14-3-3ζ (Fig. 2C, D). Compared to other pairs, the combination of 14-3-3ζ-Rluc8 and BAD-112-136F-mC yielded the most robust BRET signal (Fig. 2D).

To confirm the interactions between 14-3-3ζ-Rluc8 and BAD-112-136F-mC, we introduced single serine-to-alanine mutations at either S112 (BAD-112-136F-112A-mC), S136 (BAD112-136F-136A-mC), or a double S112/136AA mutation (BAD-112-136F-AA-mC) as these mutations are known to prevent binding of 14-3-3 proteins to BAD. We found that BAD-112-136F-136A-mC and BAD-112-136F-AA-mC could not elicit a detectable BRET signal, which likely indicated an inability for 14-3-3ζ-Rluc8 to interact with either BAD variant (Fig. 2E).

Using confocal microscopy, the subcellular localizations of 14-3-3ζ and BAD-112-136F were determined. Conjugation of (mT)-urquoise to 14-3-3ζ revealed that 14-3-3ζ is primarily restricted to the cytoplasm (Fig. 2F). We next compared the subcellular localization of BAD-mC to BAD-112-136F-mC and found that the fragment was similarly restricted to the cytoplasm. In contrast, BAD-112-136F-AA-mC was distributed throughout the cell, including the nucleus, suggesting the S-A mutations of this BAD fragment may lead to impaired sequestration by 14-3-3ζ (Fig. 2F).

**Fig. 3:**
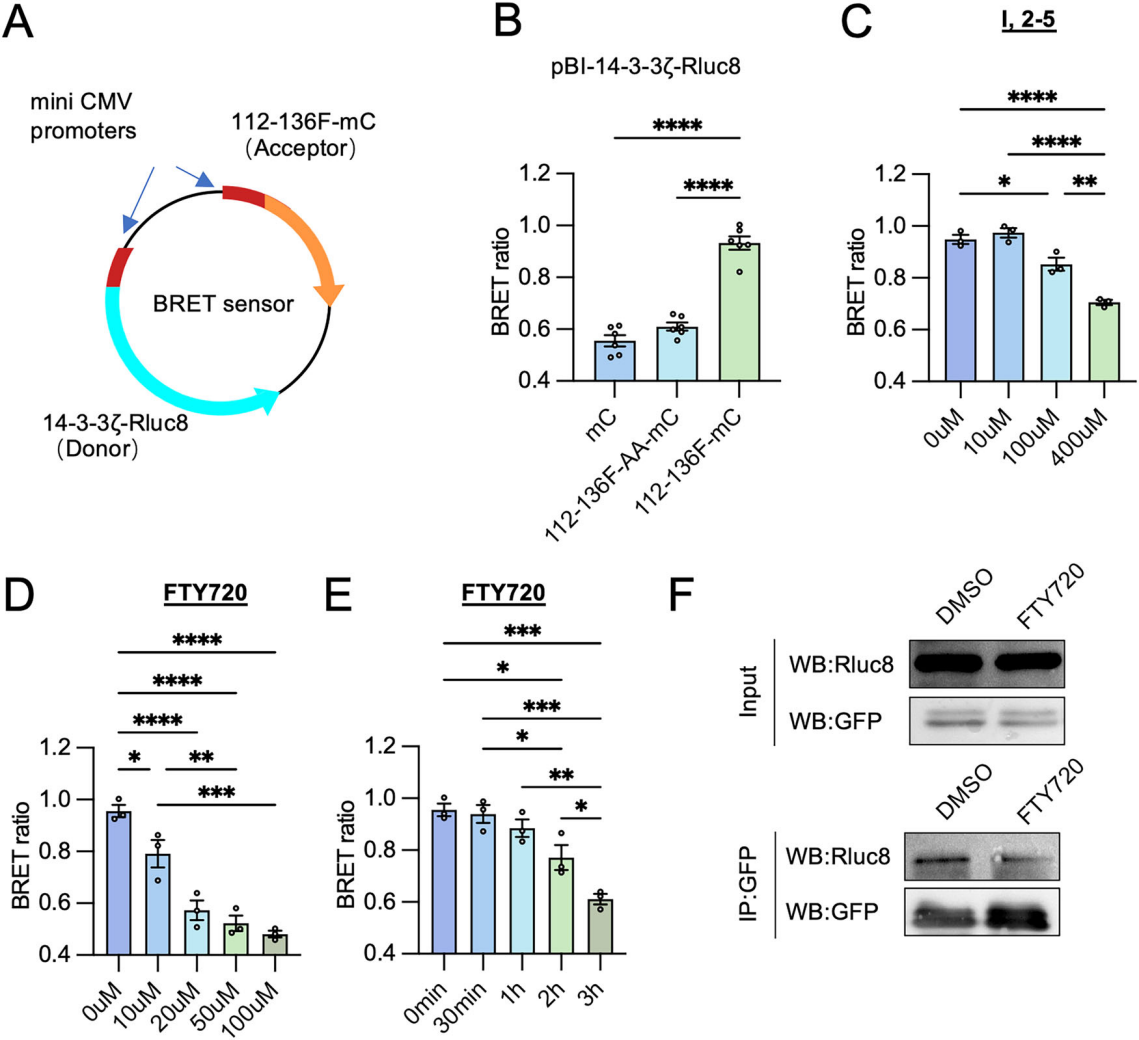
Validation of the BRET sensor. **A** Schematic of the BRET sensor design. 14-3-3ζ-Rluc8 and 112-136F-mC were inserted into pBI-CMV1 vector at MCS2 and MCS1, respectively. Plasmids with mCitrine (mC) or 112-136-AA-mC inserted in place of 112-136F-mC were generated as controls. **B** BRET ratios between 14-3-3ζ-Rluc8 and mC, 112-136-AA-mC, or 112-136F-mC were compared (*n* = 6 per group). **C** A dose-response study was conducted with I,2-5 in NIH-3T3 cells expressing the BRET sensor. Measurements were taken 3 h post-treatment (*n* = 3 per group). **D** A dose-response study was conducted with FTY720. Data were collected 3 h post-treatment (*n* = 3 per group). **E** A time-response study was conducted with FTY720 (20 µM) (*n* = 3 per group). **F** CoIP was performed to compare levels of 14-3-3ζ-Rluc8:112-136F-mC complex in DMSO-treated and FTY-720-treated BRET sensor-expressed cells. Statistical significance was determined using one-way ANOVA with Tukey’s post hoc test. *P < 0.05; **P < 0.01; ***P < 0.001; ****P < 0.0001.

With our observations that co-transfection of 14-3-3ζ-Rluc8 and BAD-112-136F-mC plasmids resulted in a detectable BRET signal, we constructed a bi-directional BRET sensor plasmid whereby the donor and the acceptor were expressed at near stoichiometric ratios due to equal promoter activities (Fig. 3A). Co-expression of BAD-112-136F-mCitrine and 14-3-3ζ-Rluc8 in NIH-3T3 cells resulted in an average of 34.56% increase in BRET compared to the co-expression of 112-136F-AA-mCitrine and 14-3-3ζ-Rluc8 (Fig. 3B). To test the performance of our bi-directional BRET sensor, we evaluated the capacity of our sensor to detect BAD-112-136F-mC:14-3-3ζ-Rluc8 interactions with two well-recognized 14-3-3 inhibitors, FTY720 and I,2-5 [49]. Treatment of sensor-expressing cells with FTY720 and I,2-5 significantly reduced BRET in a dose-dependent manner (Fig. 3C, D). To optimize conditions for the following HTS, we next determined the optimal incubation time and found that 3 h was required to reduce the magnitude of BRET to the same degree as BAD-112-136-AA, which indicated a maximal effect (Fig. 3B, E). The interaction between 14-3-3ζ-Rluc8 and BAD-112-136F-mC was further confirmed by co-immunoprecipitation. Compared to the DMSO-treated control, the abundance of 14-3-3ζ-Rluc8 within the co-immunoprecipitated complex was reduced in FTY-720-treated cells expressing the BRET sensor (Fig. 3F). These results suggest that the established BRET sensor is capable of detecting disrupted 14-3-3ζ:BAD-112-136F-mC interactions following 3-hour drug treatments and may be suitable for use in drug screening.

#### In silico modeling of BAD and 14-3-3ζ interactions confirm the performance of the BRET sensor

**Fig. 4:**
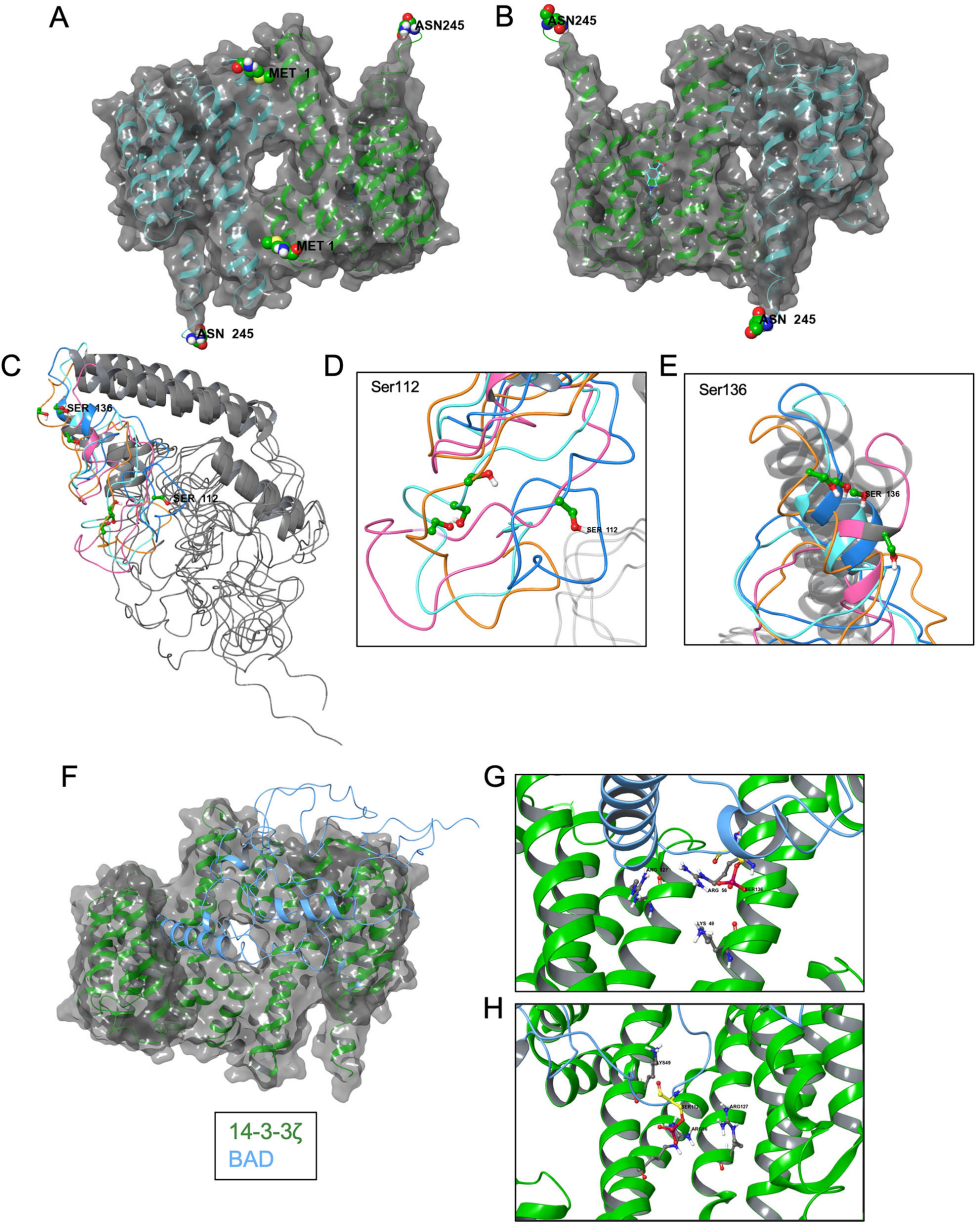
In silico analysis and modeling of interactions between 14-3-3ζ and BAD. **A**, **B** Structure of the 14-3-3ζ homodimer with one subunit shown in green ribbons and the other shown in cyan ribbons shown from (**A**) the rear with N-terminus Met1 highlighted and **B** the front with BV-02 in the binding groove shown in blue and C-terminal Asn245 highlighted. **C** Overlayed structures of full-length bad obtained from clustering GaMD simulation with Ser112 and Ser136 are highlighted in green. **D**, **E** Zoom in on Ser136 (**D**) residues of clusters and Ser112 (**E**). **F** Docked structure of full-length BAD (blue ribbons) bound to 14-3-3ζ (green ribbons) obtained from protein–protein docking. **G**, **H** Ser136 (**G**) and Ser112 (**H**) of BAD each in the amphipathic grooves of a 14-3-3ζ subunit interacting with Arg56 and adjacent to Arg127 and Lys49.

To better understand the molecular interactions between BAD or BAD-112-136F and 14-3-3ζ, in silico approaches were used. Examination of the crystal structures of 14-3-3ζ shows that the N-terminus sits at the interface of the 14-3-3ζ dimer and is located on the “rear” of the protein, opposite the binding groove (Fig. 4A). In contrast, the C-terminus is adjacent to the binding groove, potentially much closer to any BAD ligand, and this would increase the probability of interactions with mCitrine (Fig. 4B). Models of 14-3-3ζ-Rluc8, BAD-mC, and BAD-112-136F-mC were built and Gaussian accelerated MD simulations were performed to sample protein conformations. Clustering was performed to obtain representative structures. All the top clusters obtained for full-length BAD were similar and had two conserved alpha helices, but the domain between residues 112–136 is highly flexible. Ser112 and Ser136 can rotate and be inaccessible in some poses, so the topmost (lowest energy) cluster was used for further analysis as its residues were solvent exposed allowing for interactions while in some of the other clusters they were blocked (Fig. 4C–E). As a shorter peptide, and focused on the unstructured region, the BAD-fragment was significantly more flexible and may not have any actual defined structure, so the top five poses were used in the studies to sample a wide variety of possibilities (Supplementary Fig. 2A, B). Protein–protein docking was performed between 14-3-3ζ and full-length BAD and BAD-112-136F (Fig. 4F). Full-length BAD can bind with a phosphorylated serine residue in each of the aliphatic grooves of the 14-3-3ζ dimer, interacting with R56, and is adjacent to K49 and R127, which is consistent with previous reports that BAD can bind both subunits simultaneously (Fig. 4G, H) [13].

Molecular dynamic situations were then performed to compare the structures of 14-3-3ζ-Rluc8 when bound to BAD-mC or BAD-112-136F-mC. With 14-3-3ζ-Rluc8 and BAD-mC or mC-BAD, the C-termini containing mCitrine extend out to the side, away from Rluc8 on 14-3-3ζ (Supplementary Fig. 3). In contrast, the docking of BAD-112-136F-mC had mCitrine positioned much closer to Rluc8 (Supplementary Fig. 2C). The top cluster of BAD-112-136F-mC sits significantly closer to Rluc8 and binds 14-3-3ζ via S136, consistent with experimental results. Molecular dynamic simulations were also performed on full-length BAD-mCitrine bound to 14-3-3ζ-Rluc8 and the average distance between Rluc8 and mCitrine over the course of the simulations was ~80 Å based on the center of mass of Rluc8 and mCit and potentially explains the low BRET signal observed for this construct (Supplementary Fig. 4C).

#### Screening for pro-apoptotic drugs that act by disrupting 14-3-3ζ’s actions

**Fig. 5:**
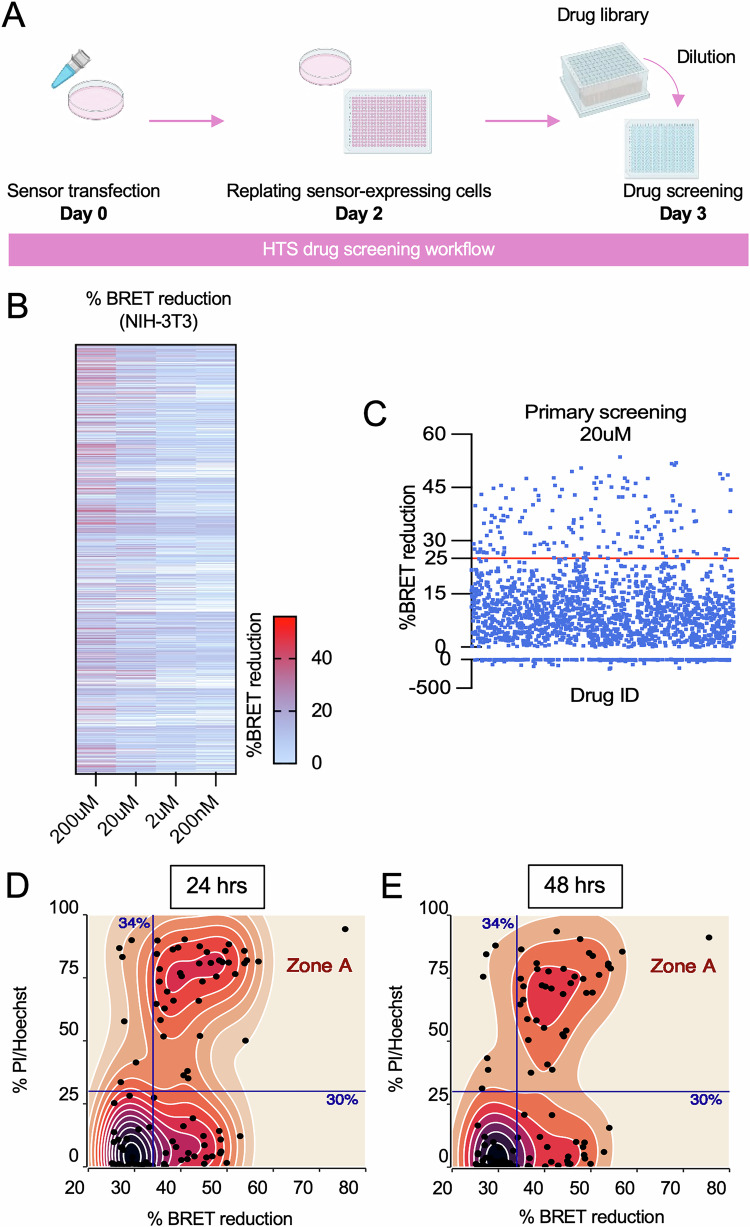
High throughput screening (HTS) for drugs that disrupt 14-3-3:BAD interactions. **A** Schematic of HTS workflow. Cells were transfected with BRET sensor or control sensors in 10 cm dishes and allowed 48 h for sensor expression. Subsequently, cells were harvested and re-plated into 384-well plates at a density of 20,000 cells per well 24 h prior to drug screening. Diluted previously approved drugs (PADs) were added to corresponding wells with final concentrations of 200 µM, 20 µM, 2 µM, and 200 nM, respectively. After a 3-hour incubation, the BRET ratio was measured Each PAD was assessed twice at each concentration, but only the highest value for each concentration was recorded. **B** The heatmap shows BRET reduction for 1971 PADs at concentrations of 200 µM, 20 µM, 2 µM, and 200 nM. **C** Subsequent screenings were conducted at 20 µM, and PADs were selected based on the median BRET reduction (26.80%) of those that demonstrated a BRET reduction greater than zero. To include the hits near this threshold, a BRET reduction of 25% was set as the selection criterion for primary screening. This led to the selection of 162 PADs for re-screening in a 96-well plate format. **D**, **E** 101 hits were further selected to assess their capacity to induce cell death in NIH-3T3 cells. Hoechst/propidium iodide incorporation assays were used to examine cell death, calculated as the ratio of propidium iodide-positive cells to Hoechst-positive cells. Cell death data were collected from three independent experiments, each with duplicate measurements. Scatter density plots are used to visualize the relationship between drug-induced BRET reduction and their capacity to induce cell death at 24 h (**D**) or 48 h (**E**) post-treatment.

The implementation of our BRET-based sensor in living cells permitted the screening of previously approved drugs (PADs) that could disrupt 14-3-3ζ:BAD-112-136F interactions (Fig. 5A). The re-purposing or re-positioning of these drugs could lead to the successful identification of new functions such as the induction of cell death. The primary screen was conducted in a 384-well plate format, and 1971 compounds were tested at 200 µM, 20 µM, and 2 µM, respectively, for 3 h (Fig. 5B). We found the median BRET reduction caused by compounds capable of reducing BRET was approximately 25%, and there were 416, 162, 31, and 16 PADs that reduced the BRET signal by 25% at concentrations of 200 µM, 20 µM, and 2 µM, respectively (Fig. 5B). To rule out the possibility that the reduction in BRET was caused by cell death rather than disrupted 14-3-3ζ:112-136F interactions, which could lead to false positive results, cell death was assessed following 3 h, and compared to 24-hour treatments, limited cell death was observed during this short incubation period (Supplementary Fig. 5). This suggests that any reductions in BRET were independent of effects on cell death.

We next adapted our screen into 96-well plates to re-screen PADs that were effective at 20 µM, a concentration commonly used in HTS assays [50]. Of these, 101 PADs showed consistent results and were further assessed for their capacity to induce cell death in NIH-3T3 cells via Hoechst/propidium iodide incorporation assays (Fig. 5C). Scatter density plots were used to visualize the relationship between BRET reduction and the induction of cell death at 24 and 48 h (Fig. 5D, E), and 41 PADs were found to reduce BRET by more than 34%, which is consistent with the reduction in BRET caused by 112-136F-AA-mCitrine (Fig. 3B), and induce cell death greater than 30% (Zone A; Fig. 5D, E). These 41 PADs were kept for in-depth investigation.

#### Inhibition of 14-3-3 proteins promotes mitochondrial translocation of BAD in CRC cells and cell death

**Fig. 6:**
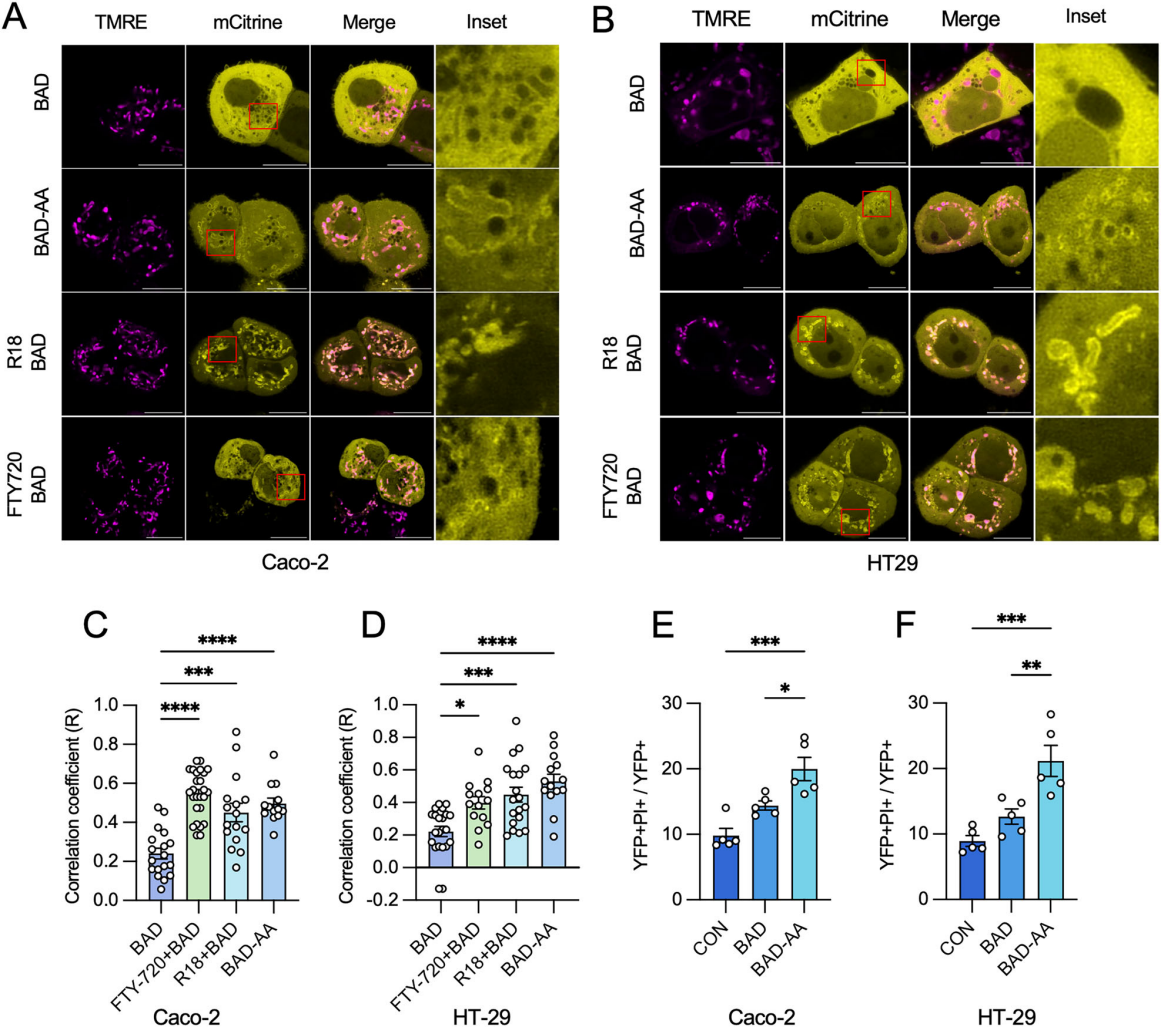
Over-expression of BAD leads to the death of colorectal cancer cells (CRCs). **A**, **B** Caco-2 (**A**) and HT-29 (**B**) were made to express mCitrine-conjugated BAD (BAD) or BAD mutants harboring S112A and S136A double mutations (BAD-AA). BAD-expressing cells were treated with either R18 (10 µM) or FTY720 (2 µM) for 24 h. The mitochondrial membrane potential sensor TMRE (100 nM) was added to the medium 1 h prior to live-cell imaging. Representative images were selected from three independent experiments, with at least five images were captured per dish in each experiment. **C**, **D** The co-localization of BAD and mitochondria in Caco-2 (**C**) and HT-29 (**D**) was assessed by calculating Pearson’s correlation coefficient (R). Each data point corresponds to a single cell. **E**, **F** Caco-2 (**E**) and HT-29 (**F**) cells were transfected with either pBI-14-3-3ζ-Rluc8-mCitrine, pBI-14-3-3ζ-Rluc8-BAD-mCitrine, or pBI-14-3-3ζ-Rluc8-BAD-AA-mCitrine and allowed for 72 h for plasmid expression. Cell death was evaluated using the Operetta CLS system. BAD-induced cell death was quantified as the ratio of YFP- and propidium iodide (PI)-double-positive cells to YFP-positive cells. Data were collected from five independent experiments, each with triplicate measurements. Statistical significance was determined using one-way ANOVA with Tukey’s post hoc test. *P < 0.05; **P < 0.01; ***P < 0.001; ****P < 0.0001.

To explore the potential of identified hits to induce apoptosis, CRC was selected as our disease model. We first determined if disruption of 14-3-3 protein:BAD PPIs could induce cell death in CRC cells. Colorectal cell lines, Caco-2 and HT-29, were transfected with either BAD-mCitrine or BAD-AA-mCitrine, followed by incubation with FTY-720 or R18. BAD translocation to mitochondria following 14-3-3 protein inhibition was similar to what was observed in NIH-3T3 cells (Figs. 1 and 6A–D). Additionally, cell death was assessed following the overexpression of BAD or BAD-AA. After 72 h post-transfection, a significantly higher degree of cell death was observed in cells co-expressing 14-3-3ζ and BAD-AA, compared to those only expressing 14-3-3ζ (control) or co-expressing 14-3-3ζ and BAD (Fig. 6E, F). These observations imply that when 14-3-3ζ is unable to sequester BAD due to S112/136A mutations or due to the presence of 14-3-3 inhibitors in CRC cells, BAD translocates to the mitochondria to induce cell death.

#### Examining the abilities of lead candidates to kill CRC cells

**Fig. 7:**
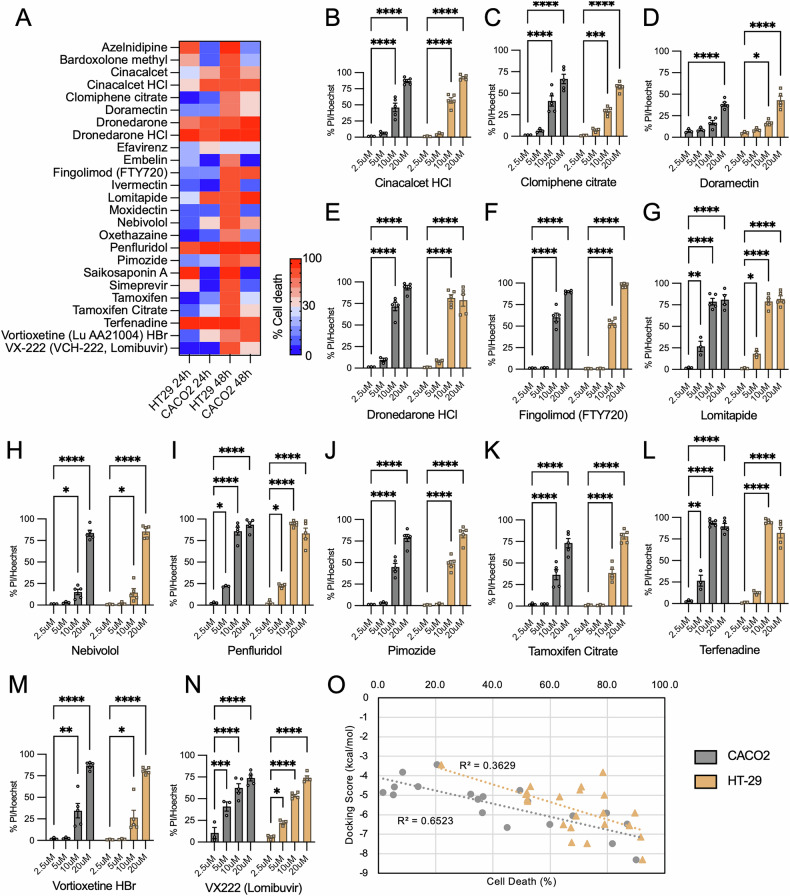
Capacity of identified hits to induce cell death in CRC cells. **A** Following the exclusion of unsuitable PADs, 25 hits were examined for their capacity to induce cell death In HT-29 and Caco-2 cells at 24 h and 48 h post-treatment, respectively. The heatmap shows that 15 of these hits induced more than 30% of cell death in both types of CRC cells. **B–N** After the filtering of pro-drugs, salts, and potency, does-response studies were performed for 13 selected hits. Lomitapide, terfenadine, penfluridol, and lomibuvir demonstrated effectiveness at concentrations as low as 5 µM. Data were collected from three (2.5 µM and 5 µM) or five (10 µM and 20 µM) independent experiments, each with duplicate measurements. **O** Correlation between docking scores (Table 2) and cell death with Caco-2 and HT-29 cells with Azelnipidine, Bardoxolone, and Saikosaponin A omitted. Statistical significance was determined using two-way ANOVA with Dunnett’s post hoc test. *P < 0.05; **P < 0.01; ***P < 0.001; ****P < 0.0001 when compared with 2.5 µM treatment.

Among the 41 identified PAD hits from our primary screen in NIH-3T3 fibroblasts, some were excluded, as they were found to be unsuitable for systemic administration. For example, crystal violet is a synthetic dye used for cell staining [51]; whereas some PADs are topical treatments, such as cetylpyridinium chloride, benzethonium chloride, and thonzonium bromide [52–54]. These are all problematic compounds, and they meet the criteria as pan-assay interference compounds (PAINS) and likely should not have been included in the library due to non-specific effects [55]. In addition, other PADs, such as entrectinib, ceritinib, and ponatinib, are already being used as chemotherapeutics, and their observed cytotoxic actions would be expected from their primary mechanisms of action [56]. After excluding these two classes of PADs, 25 were left to be assessed on Caco-2 and HT-29 CRC cells. Of these, 15 PADs caused more than 30% of cell death at 24 h or 48 h in both cell lines at 20 µM (Fig. 7A), and further filtering to remove pro-drugs, salts, and low potency compounds narrowed our list to 13 PADs. Dose-response studies were then conducted with these 13 agents (Fig. 7B–N). Lomibuvir, terfenadine, penfluridol, and lomitapide were found to be the most effective, as they significantly induced cell death at concentrations as low as 5 µM (Fig. 7G, I, L, N). Although lomibuvir consistently induced cell death at different concentrations, its efficacy in the magnitude of cell death attained was inferior to other candidates (Fig. 7N), and it was excluded from further study.

#### Observed activity of identified compounds correlates with their predicted interactions with 14-3-3

**Fig. 8:**
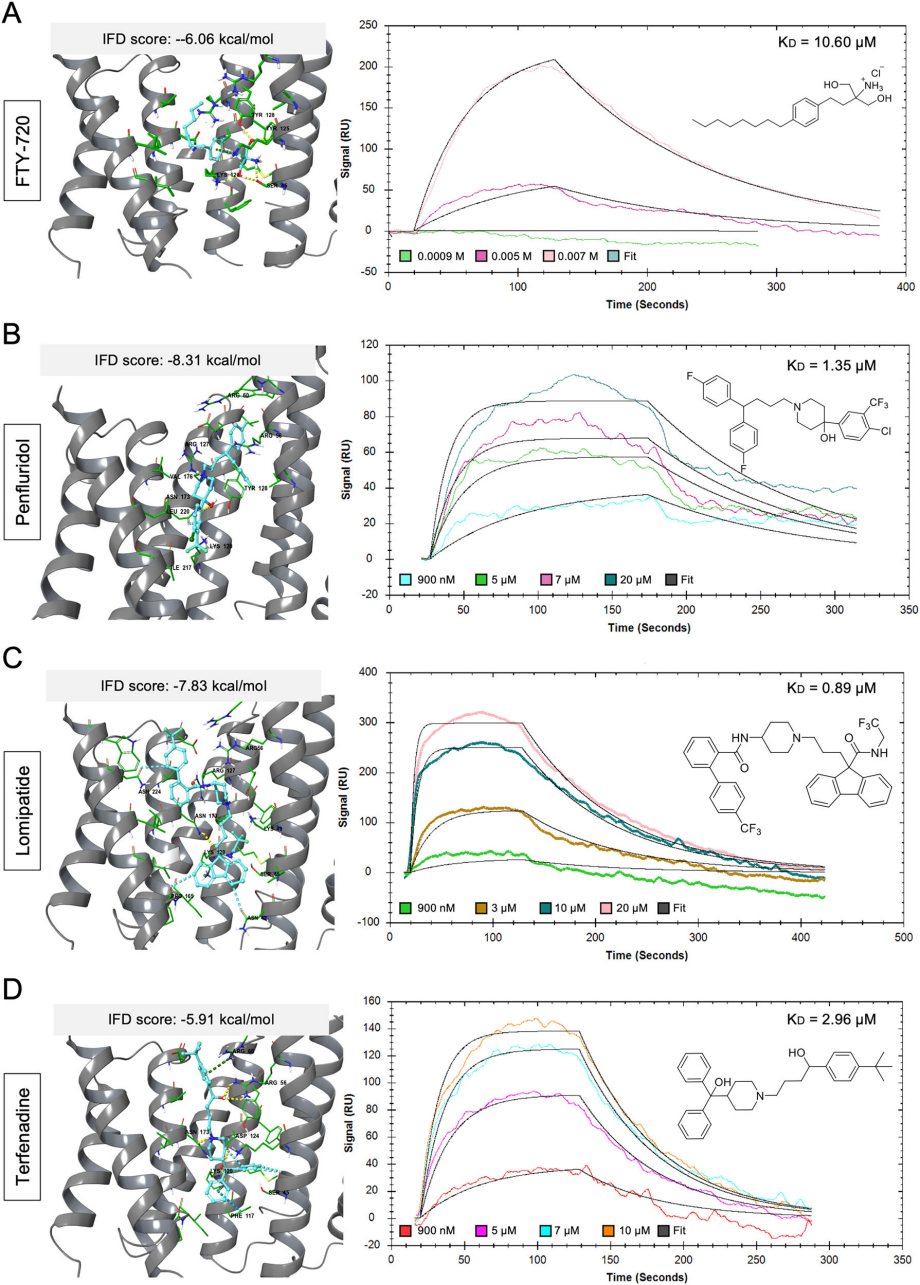
In silico and biophysical characterization of drugs with 14-3-3ζ. Induced Fit docking and surface plasmon resonance (SPR) sensograms of FTY720 (**A**), penfluoridol (**B**), lomipatide (**C**), and terfendadine (**D**). Docked structures of drugs are within the amphipathic binding groove of 14-3-3ζ (PDB: 2C1J) Residues within 4 Å are shown in green and ligands are shown in cyan. Hydrogen bonds are shown as dashed yellow lines, aromatic H-bonds as blue dashed lines, and π-cation interactions as green dashed lines. SPR assays were based on concentrations between 0.00009−0.05 mM running over an immobilized 14-3-3ζ; constant or local fitting of the association and dissociation curves (black).

We next performed induced-fit docking to explore the possible binding modes of the compounds (Table 2). BV01, BV02, and the peptido-mimetic I,2-5 were used as reference compounds, as they are known 14-3-3 inhibitors with established affinity for various 14-3-3 isoforms [46, 49, 57, 58]. Moreover, these known hits provided a good starting point for validating our approach. A fourth reference compound, FTY-720, has been previously studied against 14-3-3ζ. While no crystal structures of any of these complexes exist, the binding mode of BV02 obtained in our study matches the binding mode reported previously in similar studies, validating our preparation of the computational protein model (Supplementary Fig. 6) [58, 59]. BV02, peptide 2-5, and FTY-720 had docking scores of −6.69, −7.21, and −6.06 kcal/mol, respectively, suggesting we should observe moderate binding in a biophysical assay (Fig. 8, Supplementary Figs. 6 and 7). BV02 and peptide 2-5 are both negatively charged, and binding is largely mediated by the positively charged residues in the 14-3-3 binding groove (Supplementary Figs. 6 and 7). BV02 forms salt bridges with both R56 and R127 and additional hydrogen bonds with K49, K120, and N173. Similarly, 2-5 forms salt bridges with R56, R127, and K49 through the phosphate and additional hydrogen bonds with K120 and N173. FTY720, however, is positively charged and instead forms hydrogen bonds through the OH groups with S45, K120, Y125, and Y128 and a π-cation interaction with K49 (Fig. 8A). The interaction of FTY720 was surprising, as it has been established that FTY720 potently promotes the dissociation of 14-3-3 protein dimers [48].

**Table 2:** Cell death observed in respective cell line and docking score obtained from induced fit docking.

Compound	%Cell death		Docking score (kcal/mol)
	Caco-2	HT-29	
Azelnidipine	13.7	91.6	−7.14
Bardoxolone methyl	19.2	67.5	−7.43
Cinacalcet	49.4	70.3	−4.75
Clomiphene	34.7	52.0	−5.21
Doramectin	32.3	51.6	−4.95
Dronedarone	86.9	79.0	−6.48
Efavirenz	20.5	22.0	−3.43
Embelin	1.7	51.8	−4.86
FTY720	67.9	69.1	−6.06
Ivermectin	5.2	78.9	−4.98
Lomitapide	81.7	72.9	−7.48
Moxidectin	8.4	78.4	−3.82
Nebivolol	45.0	67.3	−6.66
Oxethazaine	13.9	53.1	−4.55
Penfluridol	90.0	92.2	−8.31
Pimozide	59.9	64.6	−6.5
Saikosaponin A	1.2	87.6	−6.56
Simeprevir	5.5	70.9	−4.57
Tamoxifen	36.5	63.3	−5.07
Terfenadine	79.7	89.6	−5.91
Vortioxetine	52.1	52.1	−5.31
VX-222 (VCH-222, Lomibuvir)	36.3	78.5	−5.9

Despite the preference for negatively charged ligands, Penfluoridol, Lomitapide, and Terfenadine have good docking scores (−8.31, −7.48, and −5.91 kcal/mol, respectively), though this is largely driven through aromatic H-bonds and π-cation interactions rather than salt-bridge formation (Fig. 8B–D). Penfluridol forms a hydrogen bond with N173 as well as an aromatic H bond, and an aromatic H bond with E131. It could also potentially form several π-cation interactions with adjacent R58, R60, and R127, however, this was not observed due to the limited flexibility in IFD. Terfenadine can form several H-bonds with R56, N173, K120, and D120 as well as a π-cation with R60, aromatic H bonds with S45 and D124, and a π-π stacking interaction with P117. From the compounds examined, Penflridol, and Lomitapide had the best docking scores which correlated with observed experimental results where they induced the most cell death (Table 2; Fig. 7G, I). Bardoxolone and azelnidipine also had good docking scores, while terfenadine was lower at −5.91 kcal/mol (Table 2). Generally, with the exception of Azelnipidine, Bardoxolone, and Saikosaporin A, compounds that had similar or better docking scores than FTY-720 strongly induced cell death in Caco-2 cells after 48 h and docking scores correlate well with observed cell death (Fig. 7O). Some compounds, such as cinacalcet (−4.75 kcal/mol), Clomiphene (−5.21 kcal/mol), and doramectin (−4.95 kcal/mol), had weaker docking scores and were found to induce moderate levels of cell death. Overall, the docking score appears to be a good predictor of whether compounds were capable of inducing cell death in Caco-2 cells.

#### Cell-free biophysical characterization of binding of the lead PADs with purified 14-3-3ζ

Recombinant His-14-3-3ζ was generated and purified to the perform biophysical assays (Supplementary Fig. 8). Despite significant effort, we were unable to obtain convincing evidence of target engagement with any of the tested compounds using isothermal titration calorimetry (ITC, Supplementary Fig. 9), although a binding event is suggested for the final two compounds. The challenge arises from an unfortunate combination of factors: 14-3-3ζ is unstable in more than 4% DMSO (v/v), while the drugs are all highly hydrophobic with poor solubility at physiological ionic strength that require higher levels of DMSO to be fully soluble. In 4% DMSO, nanoaggregates, as measured by dynamic light scattering, were formed, despite achieving concentrations high enough to elicit a signal due to their only moderate affinity for the protein.

Surface plasmon resonance (SPR) was then employed. His-14-3-3ζ was immobilized onto a SPR chip with the ligands being introduced in the running buffer (Supplementary Fig. 10). BV01 and BV02 were included as controls, as they were designed to directly engage with 14-3-3ζ. We conducted dynamic light scattering-experiments to accurately set the upper limit of concentrations to avoid nanoaggregations, and we found concentrations of 9 nM to 50 mM to be the safest range to work in. Testing of BV01 and BV02 against immobilized 14-3-3ζ resulted in K_D_ values of 6.32 μM and 90.10 μM, respectively (Supplementary Fig. 6), and this established conditions to test our 3 hit leads and reference drug (Fig. 8). K_D_ values in the μM range for FTY720 (10.60 μM), Penfluoridol (1.35 μM), Lomitapide (0.89 μM), and Terfeindine (2.96 μM) were determined. The complete tables of association and dissociation rates are provided as Supplementary Table 2 and the relationships between drug concentrations and binding affinities were also explored (Supplementary Fig. 11). Negative decoy controls showed no meaningful binding, suggesting that the observed effect is specific for the protein-ligand pairs (data not shown). These values are broadly aligned with what one would expect based on the computational induced-fit docking scores; all compounds are good ligands for the protein as suggested by the BRET assay.

#### Verifying the capacity of terfenadine, penfluridol, and lomitapide to induce apoptosis in cancer cells

We first attempted to determine if the abilities of our lead candidates to induce cell death in Caco-2 and HT-29 cells were dependent on BAD activity, through the use of siRNA-mediated depletion (Supplementary Fig. 12). Moderate siRNA-associated knockdown of BAD was only achievable in Caco-2 cells, and despite achieving reductions in BAD abundance, our lead candidates were still able to induce cell death (Supplementary Fig. 12D, E). The inability to significantly reduce BAD abundance could be due to cellular resistance to transfection with siRNA or high rates of cell proliferation, both of which would impact the interpretation of our findings.

**Fig. 9:**
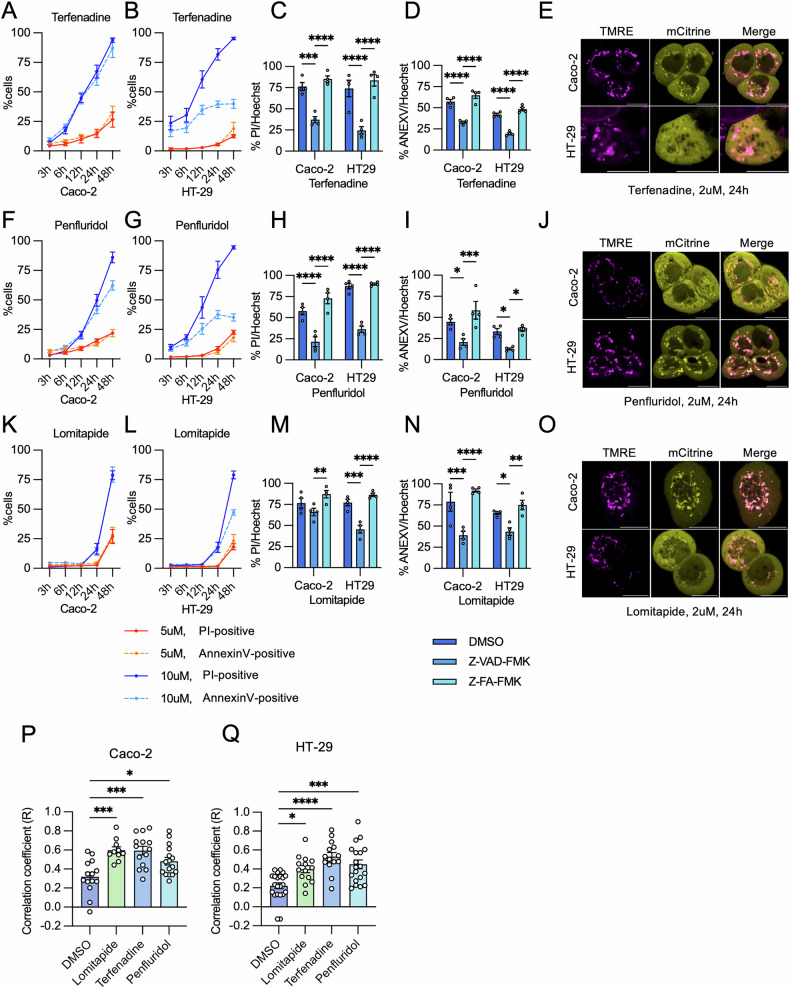
Ability of lead hits to induce apoptotic cell death in colorectal cancer cells. **A**, **B**, **F**, **G**, **K**, **L** Time-response studies using Hoechst/propidium iodide (PI)/annexin V (ANEXV) incorporation assays were conducted for terfenadine (**A**, **B**), penfluridol (**F**, **G**), and lomitapide (**K**, **L**) in Caco-2 (**A**, **F**, **K**) and HT-29 (**B**, **G**, **L**) cells at concentrations of 5 µM and 10 µM. Data were collected from three (5 µM) or five (10 µM) independent experiments, each with duplicate measurements. **C**, **D**, **H**, **I**, **M**, **N** The pan-caspase inhibitor Z-VAD-FMK or its control inhibitor Z-FA-FMK (both at 20 µM) was introduced to determine the mechanism of cell death induced by lead PADs. PI incorporation (**C**, **H**, **M**) and annexin V incorporation (**D**, **I**, **N**) were measured in Caco-2 or HT-29 cells after 24 h (**C**, **D**, **H**, **I**) or 48 h (**M**, **N**) of treatment. Data were collected from four independent experiments, each with duplicate measurements. **E**, **J**, **O** Mitochondrial translocation of BAD was observed in CRC cells treated with terfenadine (**E**), penfluridol (**J**), and Lomitapide (**O**) at 2 µM, 24 h post-treatment. Representative images were selected from three independent experiments, with three images were captured per dish in each experiment (Scale bar = 10 µm). **P**, **Q** The co-localization of BAD and mitochondria in Caco-2 (**P**) and HT-29 (**Q**) cells treated with terfenadine (**E**), penfluridol (**J**), and Lomitapide (**O**) was assessed by calculating Pearson’s correlation coefficient. Three images were captured per dish from three independent experiments, and each data point corresponds to a single cell. Statistical significance was determined using two-way ANOVA with Tukey’s post hoc test. *P < 0.05; **P < 0.01; ***P < 0.001; ****P < 0.0001.

Cell death can emerge from a variety of different pathways: including apoptosis, necrosis, and autophagy [1]. Given that caspase activation is a hallmark of apoptosis, a pan-caspase inhibitor, Z-VAD-FMK, was used to attenuate hit PAD-induced apoptosis [60]. According to time courses of PI and Annexin V incorporation (Fig. 9A, B, F, G, K, L), differences in the kinetics of cell death or apoptosis were seen with the PAD hits in HT-29 and Caco-2 cells, such that terfenadine and penfluridol were able to induce cell death and apoptosis more rapidly than lomitapide. To assess caspase-dependent apoptosis, HT-29 and Caco-2 cells were treated for 24 h with terfenadine (10 μM) (Fig. 9C, D) and penfluridol (10 μM) (Fig. 9H, I), or for 48 h for lomitapide (10 μM) (Fig. 9M, N), along with either Z-VAD-FMK or its control, Z-FA-FMK [61]. Significantly diminished lead PAD-induced propidium iodide and/or annexin-V incorporation were observed in cells pre-treated with Z-VAD-FMK compared to those pre-treated with either DMSO or a control inhibitor Z-FA-FMK, implying caspase activation following drug administration. We next assessed if cell death induced by our lead PADs was associated with mitochondrial translocation of BAD upon drug exposure, and confocal imaging showed a redistribution of BAD to mitochondria after the addition of the drugs (Fig. 9E, J, O, P, Q), demonstrating that disruption of 14-3-3ζ with lead PADs induces apoptosis likely via the translocation of BAD to mitochondria.

## Discussion

Cancer arises from uncontrolled cell proliferation—one of the primary mechanisms of chemotherapies is to induce cell death in proliferating cells. The overexpression of 14-3-3ζ and its related isoforms in the context of cancer has long been associated with poor clinical outcomes due to increased cell survival in the face of chemotherapeutic treatment [16, 62]. Although inhibiting 14-3-3ζ triggers apoptosis in cancer cells, there are currently no approved therapeutics that target 14-3-3ζ:BAD interactions [18]. The primary aim of this study was to explore the possibility of identifying compounds by their capacity to interrupt 14-3-3ζ:BAD interactions, but for this to be achieved, a suitable, mechanistically specific assay is needed and was not available. Thus, we created this required tool by innovating a biosensor capable of detecting 14-3-3ζ:BAD PPIs. Importantly, our BRET-based biosensor was capable of detecting 14-3-3ζ:BAD PPIs in living cells, which provides physiological relevance.

In this study, a short fragment of murine BAD, BAD-112-136F, was used to construct the BRET sensor in place of full-length BAD. Previous research has demonstrated that BAD overexpression leads to apoptosis in various cell types [63, 64], and an advantage of using the BAD-112-136F is the absence of the BH-3 domain, which prevents the fragment from interacting with pro-survival BCL-2 proteins to promote apoptosis [65]. Furthermore, it was not possible to detect BRET when the Rluc8 acceptor and mCitrine donor were fused to 14-3-3ζ and full-length BAD, respectively. Since energy transfer between Rluc8 and mCitrine requires a distance shorter than 10 nm, we assumed that the inability to detect BRET was due to the distance between the fusion sites and interaction sites. In reported crystal structures, the N-terminus of 14-3-3ζ is positioned on the rear of the protein on the opposite side of the aliphatic groove and sits on the interface of the 14-3-3ζ [36]. This could potentially interfere with dimer formation and function, and due to its location, Rluc8 could be blocked from interacting with the mCitrine attached to bound ligands. Additionally, our modeling suggests that when full-length BAD-mCitrine is bound to 14-3-3ζ, mCitrine is positioned far away from Rluc8 at the end of a flexible tether. This increased distance, and the low occupancy of any state within the BRET distance to Rluc8, predicts that there would be no meaningful BRET signal. TR-FRET, a technique where a fluorescent protein is used as an energy donor instead of luciferase, has been previously used to screen for disruptors of 14-3-3:BAD PPIs, but a limitation was the fusion of the FRET acceptor to the serine residue that mediates interactions [27]. Thus, we chose not to pursue this screening modality as it would directly interfere with the needed binding mode and potentially generate false positives. Instead, different truncated forms of BAD were generated to identify the optimal fusion strategy for measuring BRET efficiency between 14-3-3ζ-Rluc and mCitrine-conjugated BAD variants. In our modeling of BAD-112-136F-mC, we saw that the fragment could bind 14-3-3ζ with mCitrine positioned much closer to Rluc8 than in full-length BAD. Although the peptide is more flexible and allows the fluorophore to move through a greater range, almost all the lowest energy conformations keep the Rluc8 and mCitrine within the necessary BRET distance, leading to a strong signal upon binding. Given that the interactions between 14-3-3ζ and BAD occur between the C-terminus of 14-3-3 and S112 or/and S136 of BAD, it was not surprising that the combination of 14-3-3ζ-Rluc8 and 112-136F-mCitrine represented the optimal combination in the constructing the BRET sensor [13, 66].

Since it was uncertain if the BAD-112-136F could represent the full-length BAD in its interactions with 14-3-3ζ, further evaluations were conducted by introducing mutations at Ser-Ala mutations at S112 and/or S136. Unlike the S112A mutation, S136A significantly disrupted the association between 14-3-3 and BAD-112-136F, as indicated by reduced BRET. This aligns with a prior study suggesting that S136, rather than S112, primarily mediates 14-3-3ζ:BAD interactions [67]. This hypothesis is completely in line with our computational modeling as S136 on BAD engages in important H-bonds with R56, while S112 appears to merely form a weaker electrostatic interaction with R127 and K49 of 14-3-3ζ. We see no meaningful predicted difference in these key binding motifs between full-length BAD and the truncated versions from the in silico calculations. Additionally, in contrast to 112-136F-AA, 112-136F is specifically sequestered in the cytoplasm. This indicates that 14-3-3ζ interacts with this truncated form similarly to how it would interact with full-length BAD, but only if the serine residues crucial for 14-3-3:BAD interactions remain intact.

To assess the capacity of our BRET sensor to detect disruptions of 14-3-3ζ:BAD PPIs, we introduced two well-known 14-3-3 inhibitors, FTY720 and I-2,5, and both compounds significantly reduced BRET. It is worth mentioning that we did not assess if there were any differences in affinity between 14-3-3ζ:BAD and 14-3-3ζ:112-136F. Nevertheless, drugs identified to disrupt 14-3-3ζ:112-136F PPIs should be as effective, as this smaller fragment likely accesses the binding groove of 14-3-3ζ more readily than the full-length BAD [68]. Additionally, it is worth mentioning that the high homology among different 14-3-3 isoforms permits them to share client proteins and form homo- or hetero-dimers, suggesting that the identified PADs are highly likely to disrupt PPIs not only between BAD and 14-3-3ζ but also between BAD and other 14-3-3 isoforms [66]. An important caveat of our reporter system is that we cannot experimentally distinguish if PADs directly block or disrupt the amphipathic groove of 14-3-3ζ where PPIs occur, or if the PADs act by promoting the dephosphorylation of Ser112 and Ser136F on the BAD fragment [11, 63, 64]. However, our in silico calculations and biophysical measurements strongly suggest that our lead candidates, for the most part, work through direct competitive target engagement, as indicated by the results of the biophysical SPR assays. Crystallography or covalent trapping studies will be needed to define the binding mode of the individual PADs with 14-3-3ζ.

The efficiency of utilizing HTS to develop novel anti-cancer compounds has been underscored by the discovery of sorafenib, palbociclib, and ABT-199 [69–72]. However, a recognized drawback of this drug discovery approach is the increased risk of false positives and false negatives due to the lack of replication and the use of miniaturized reaction systems [73, 74]. To increase the chances of identifying potential compounds, we first ensured the robustness of our sensor by achieving a Z-factor greater than 0.5 [34]. Second, to minimize false negatives, we tested each compound twice at four different concentrations in our primary screens but only recorded the highest BRET reduction for each concentration. After evaluating the capacity of identified compounds to induce cell death in NIH-3T3 fibroblasts, a group of drugs that decreased BRET by more than 34% and triggered more than 30% of cell death emerged as potential hits capable of killing target cells by disrupting 14-3-3ζ:BAD PPIs. Interestingly, this BRET reduction aligns with that caused by 112-136F-AA-mCitirine, suggesting that the ability of a compound to completely dissociate the 14-3-3ζ:112-136 F complex is indicative of its potential to induce cell death. Another group of drugs that were capable of reducing BRET reduction by more than 34%, but without notable efficacy in inducing cell death in NIH-3T3 cells, arose from our screens. A possible explanation for this is that our screening is based on a cell-based assay [50], and the complex intracellular environment makes it challenging to determine whether the dissociation of 14-3-3ζ:BAD resulted from a direct inhibitory action on 14-3-3ζ or BAD, or an indirect effect on the upstream signaling pathways that promote 14-3-3ζ:BAD interactions, or possibly a general mechanism of interference of the assay through non-specific absorption to Rluc8. Therefore, other than disrupting 14-3-3:BAD interactions, these compounds may have additional effects, such as up-regulating anti-apoptotic BCL-2 proteins, which promote cell survival [12].

Although the altered expression of 14-3-3ζ in CRC has been reported in several studies, the role of 14-3-3ζ:BAD PPIs in the survival of CRC cells remains unclear [18, 75, 76]. Our research provides the first evidence that disruption of 14-3-3ζ:BAD PPIs can promote CRC cell death. We used two representative CRC cell lines, Caco-2 and HT-29, to validate our assay [77–79]. Terfenadine, penfluridol, and lomitapide were advanced as the most promising hits after conducting dose-response studies with the 13 most potent compounds.

To support our experimental work, we modeled the possible binding modes of hits to 14-3-3ζ and compared them with the most likely binding modes of known inhibitors BV-02 and I,2-5. All compounds are capable of fitting the aliphatic groove but had varying docking scores, and many were predicted to only have moderate affinity. None of the compounds were predicted to have nM affinity based on the docking scores. Compounds BV-02 and I-2,5 were designed to take advantage of the phosphate binding region which contains numerous Arg and Lys residues and have their binding largely driven by the formation of hydrogen bonds and salt bridges. However, for many screened compounds, aromatic H-bonds and π-cation interactions played a significant role, especially in top compounds Penfluridol and Lomitapide. Interestingly, the top two compounds experimentally, penfluridol and lomitapide, had the best docking scores. Docking scores also align well with observed Caco-2 cell death at the 48-hour mark and better docking scores correlated with higher levels of cell death. Exceptions were Azelnipidine, Bardoxolone methyl, and Saikosaponin A; however, as these did induce cell death in HT29 cells but are not predicted to have strong binding, this may be due to completely separate cytotoxic mechanisms that are independent of 14-3-3ζ and BAD [80–83]. Surprisingly, some compounds also had similar or better docking scores than known 14-3-3 protein inhibitors, which suggested that they indeed bound to 14-3-3ζ. We also note that this computational model does not account for any variability in cell permeability, localization, cell-driven degradation, or more importantly off-target effects. Corrections for these features likely would improve linearity, and it must be remembered that all compounds in the assay are existing drugs with established biological activity through target engagement with other proteins. Despite these caveats, the model still shows predictive power in separating effective from less effective compounds that are all active over a tight range. This suggests that both the model is likely reasonable and that the observed cell toxicity is at least partially ascribable to this mechanism, rather than due to the known other activity of these compounds.

These predictions held up when a selection of the compounds were evaluated using an SPR assay. As suggested by the computational screen, the compounds had single digit μM dissociation constants (with lomipatide measured at 900 nM, and FTY-720 at 10.6 μM), and both tefenadine (2.96 μM) and penfluridol (1.35 μM) both also showing better affinity than the established 14-3-3 binder, BV01. Data from BV02 was too noisy to be interpreted with any confidence, consistent with reports of its tautomerization and instability and the difficulty in interpreting its SPR spectra [58, 84, 85]. These compounds show the best ever measured affinity for small molecule binders of 14-3-3ζ, highlighting their potential as excellent starting points for the development of new binders, and the success of this workflow to identify hits.

Additionally, we also collected data on the efficacy of all 101 compounds that were identified from the primary screen in inducing cell death in these two CRC cell types at 20 μM (Supplementary Table 1). These data would be invaluable for future research exploring the different capacities of drugs to target CRC cells and provide the essential information needed to initiate a rational drug design campaign starting from any of these hits.

To confirm that lead PADs induce apoptotic cell death, we further tested whether inhibiting caspase activation could mitigate lead PAD-induced cell death and if these lead PADs could promote the mitochondrial translocation of BAD. In most cases, Z-VAD-FMK treatment prevented increases in PI-positive and Annexin-V-positive cells; however, in lomitapide-treated Caco-2 cells, Z-VAD-FMK had no effect on propidium iodide incorporation, despite preventing annexin V incorporation (Fig. 8M). This is likely due to the kinetics of lomitapide in Caco-2 cells, whereby the early stages of apoptosis, marked by the binding of annexin V to phosphatidylserine, is being observed without a loss of membrane integrity that is needed for propidium iodide entry into the cell [86]. As Z-VAD-FMK cannot inhibit necroptosis, a process where cells shift to necrosis when they cannot complete apoptosis [87, 88], other forms of cell death may also be occurring. Nevertheless, confocal imaging showed that all three lead PADs trigger the mitochondria translocation of BAD, confirming apoptotic cell death.

Our study primarily focused on identifying compounds with the ability to disrupt interactions between 14-3-3ζ and BAD, but the converse of screening for compounds that either promote the association of 14-3-3ζ with BAD or those that stabilize the interaction are theoretically possible. This alternative approach is supported by recent studies focusing on interactions between 14-3-3 proteins and TAZ, a downstream effector of the Hippo pathway that regulates cell proliferation and apoptosis [89]. Activation of the Hippo pathway leads to phosphorylation of TAZ, a transcriptional co-activator, resulting in its sequestration in the cytoplasm by 14-3-3 and subsequent degradation, which inhibits cell proliferation. Inhibition of the Hippo pathway has been associated with carcinogenesis. To identify modulators of the 14-3-3:TAZ interaction, a NanoLuc® Binary Technology (NanoBiT)-based assay was developed and adapted for HTS [90]. In another HTS study, 14-3-3 proteins were used in a NanoBRET-based assay to screen for molecular glue compounds capable of stabilizing 14-3-3 interactions with various client proteins, including TAZ [91]. The development of alternative HTS assays that focus on 14-3-3ζ and BAD interactions may lead to the discovery of novel chemotherapies.

With the variability of 14-3-3 protein expression across individuals, a personalized medicine approach could one day be undertaken to explore the potential of lead PADs to treat CRC by careful evaluation of tissues from people living with CRC [76]. A significant advantage of our screening strategy is our focus on repurposing drugs from PADs. Therefore, all of our identified hits have been tested for their safety in at least prior phase 1 clinical trials. Interestingly, penfluridol and lomitapide have been previously suggested to have potential for treating CRC, but their mechanisms were not fully defined—their designed mechanisms of action are not related to driving cell death [92–94]. Our study not only further validates their therapeutic value but also provides insight into the mechanism by which these drugs may ameliorate CRC. Nevertheless, we acknowledge that the anti-cancer effects of our hit compounds on CRC cell lines may not directly translate to clinical efficacy. Although the concentrations used in our screening and cell death assays are consistent with those reported in other in vitro studies [92, 94, 95], additional in-depth preclinical studies in animal models are necessary to evaluate their pharmacokinetics, systemic tolerability, and therapeutic index as anti-CRC agents. We also recognize that PADs may also have effects on other cell types, and improving cell type specificity is clearly warranted [9]. A potential approach could also be to adopt a localized use of PADs to treat colorectal tumors, whereby intratumural drug administration might also enhance the specificity of these compounds [96].

With the use of a novel BRET-based sensor to monitor 14-3-3ζ:BAD interactions in living cells, we successfully identified terfenadine, penfluridol, and lomitapide as having the ability to disrupt 14-3-3ζ:BAD interactions and induce apoptosis of CRC cells. Although further research is critical to validate the ability of these compounds to ameliorate CRC in animal models and in humans, these hits may represent potential chemical backbones that may one day be modified and translated into new chemical entities for the treatment of CRC. This screening approach: incorporating a primary BRET screen, secondary cytotoxicity and apoptosis screens, and a tertiary biophysical confirmation, can be further leveraged by using a preliminary ultra-high throughput virtual screen to examine a far larger chemical space. As it was able to identify four compounds with higher affinity than the established 14-3-3 binders, and that all compounds advanced to the biophysical assay were demonstrated to have low μM dissociation constants. This workflow shows significant potential for the discovery of novel therapeutics for the treatment of other 14-3-3ζ apoptosis-related diseases.

## Supplementary information

Supplemental Figures and Information

Supplemental Table 1

Supplemental Table 2

## Data Availability

Computational co-ordinates, including the docking poses, prepared proteins and peptides, and representative frames from the MD simulations are available from the Borealis Dataverse, a repository jointly operated by the Canadian Universities and Research Institutes, at 10.5683/SP3/IT6KHN. Original FIDs associated with the NMR spectra for the synthesis of BV01, and both reports of the ITC and SPR runs discussed in the article, and the raw files from the instrument for those experiments, are available for download and reanalysis from the Trant Team Dataverse, hosted on the Borealis Dataverse at: 10.5683/SP3/XAIW2S.
