# Supplementary material for: A high-throughput screening approach to discover potential colorectal cancer chemotherapeutics: repurposing drugs to identify novel disruptors of 14-3-3 proteins
Source: Cell Death Dis. 2025 Nov 10;16(1):825. doi: 10.1038/s41419-025-08150-6 (PMC12603070; doi:10.1038/s41419-025-08150-6)
Supplement: Supplementary file 1 — Supplemental Figures and Information [file 41419_2025_8150_MOESM1_ESM.pdf]

Supplementary Information for:

A high-throughput screening approach to discover potential colorectal cancer chemotherapeutics: Repurposing drugs to identify novel disruptors of 14-3-3 proteins

Siyi He[1,2], Daniel Meister[3], Samra Khan[3–], Azam Mohammadzadeh[3], Luis Delgadillo Silva[2], Guy A. Rutter[1,2,4,5,6], John F. Trant,[3,7,8,9] Gareth E. Lim[1,2*]

[1]Department of Medicine, Faculty of Medicine, Université de Montréal, Montréal, QC, Canada

[2]Cardiometabolic axis, Centre de Recherche du Centre hospitalier de l'Université de Montréal (CRCHUM), Montréal, Québec, Canada.

[3] Department of Chemistry and Biochemistry, University of Windsor, 401 Sunset Ave. Windsor, ON, N9B 3P4 Canada

[4] Department of Diabetes, Endocrinology and Medicine, Faculty of Medicine, Imperial College, London, UK.

[5] LKC School of Medicine, Nanyang Technological College, Singapore, Republic of Singapore.

[6] Research Institute of the McGill University Health Centre, Montreal, Canada

[7] WE-Spark Health Institute, 401 Sunset Avenue, Windsor, ON, N9B 3P4, Canada

[8] Binary Star Research Services, LaSalle ON, N9J 3X8, Canada

[9] Department of Biomedical Sciences, University of Windsor, 401 Sunset Ave. Windsor, ON, N9B 3P4 Canada

*** Correspondence:** Gareth E. Lim, Ph.D., CRCHUM, Tour Viger, Rm 08.482, 900 Rue St. Denis, Montréal, QC H2X 029, Canada; gareth.lim@umontreal.ca; Tel: (514) 890-8000 ext 12927.

Table of Contents

Supplemental Figures .... **Error! Bookmark not defined.**

Supplemental Figure 1. Truncated forms of BAD used in the development of the BRET sensor. 3

Supplemental Figure 2. Modelling of BAD-112-136F-mCit and docking to 14-3-3ζ-Rluc8.4

Supplemental Figure 3. Docking of Full length BAD-mCit and mCit-BAD to 14-3-3ζ-Rluc8. 5

Supplemental Figure 4 Analysis of MD simulations.. 6

Supplemental Figure 5. The period of time used for BRET measurements is not sufficient to induce cell death.. 7

Supplemental Figure 6. Docking and SPR data for BV01 and BV02 suggest specific binding to 14-3-3ζ.. 8

Supplemental Figure 7. Induced fit docking of 1,-2-5 with 14-3-3ζ.................................. 9

Supplemental Figure 8. Purification of recombinant His-14-3-3ζ.................................. 10

Supplemental Figure 9. Insoluble nature of tested compounds resulted in poor application to isothermal titration calorimetry (ITC) studies. 11

Supplemental Figure 10. HS-NTA sensor activation and protein immobilization for SPR measurements.. 12

Supplemental Figure 11. Surface plasmon resonance relationship between concentration and signal response of reference compounds and lead candidates. 13

Supplemental Figure 12. Depletion of BAD in CRC cells does not affect the ability of lead candidates to induce cell death.. 14

Synthetic Experimental Details 15

Supplemental Scheme 1. Scheme for the chemical synthesis of BV01 15

A. Specific protocols for the synthesis of BV01 and starting materials. 15

A1. Synthesis of 4-chloro-3,5-dinitrobenzoic acid (1) 15

A2. Synthesis of hexyl 4-aminobenzoate (2) 16

A3. Synthesis of 4-((4-((hexyloxy)carbonyl)phenyl)amino)-3,5-dinitrobenzoic acid (BV01) 16

Supplemental Figure 13. ^{1}H NMR spectrum of Compound 1 in DMSO-d_6 18

Supplemental Figure 14. ^{1}H NMR spectrum of Compound 2 in DMSO-d_6 19

Supplemental Figure 15. ^{1}H NMR spectrum of BV01 in MeOD-d_4 20

References 21

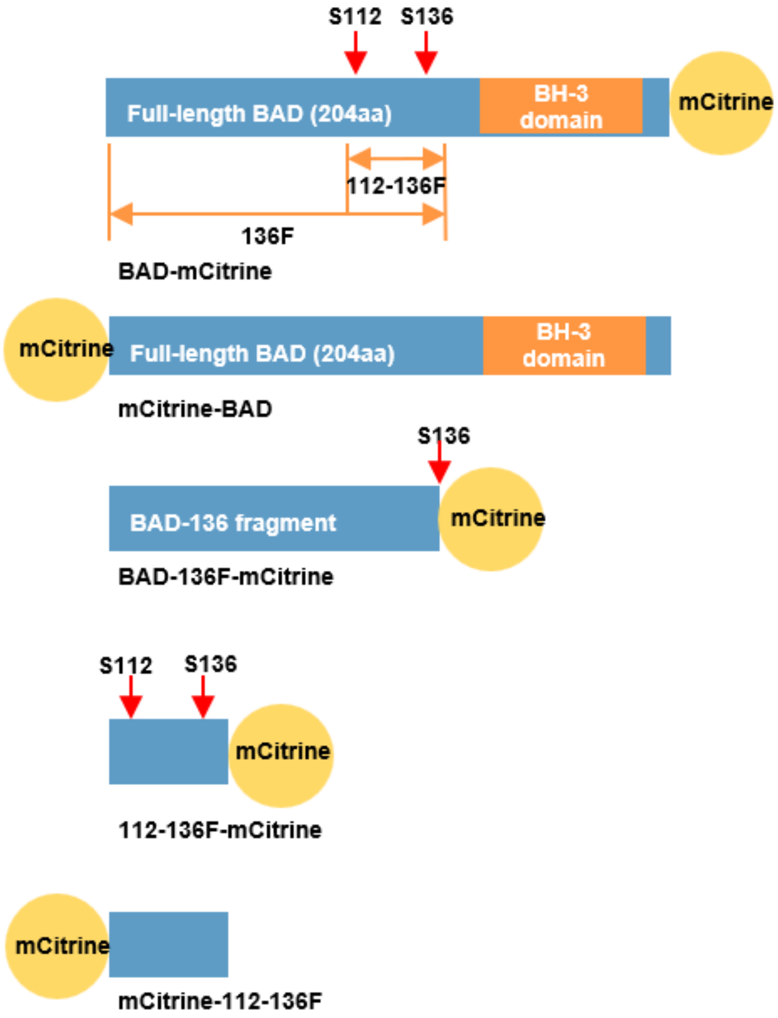

Supplemental Figure 1. Truncated forms of BAD used in the development of the BRET sensor.

Schematic of the various truncated forms of BAD that were generated during the development of the bi-directional 14-3-3ζ-Rluc8 and 112-136F-mC BRET sensor.

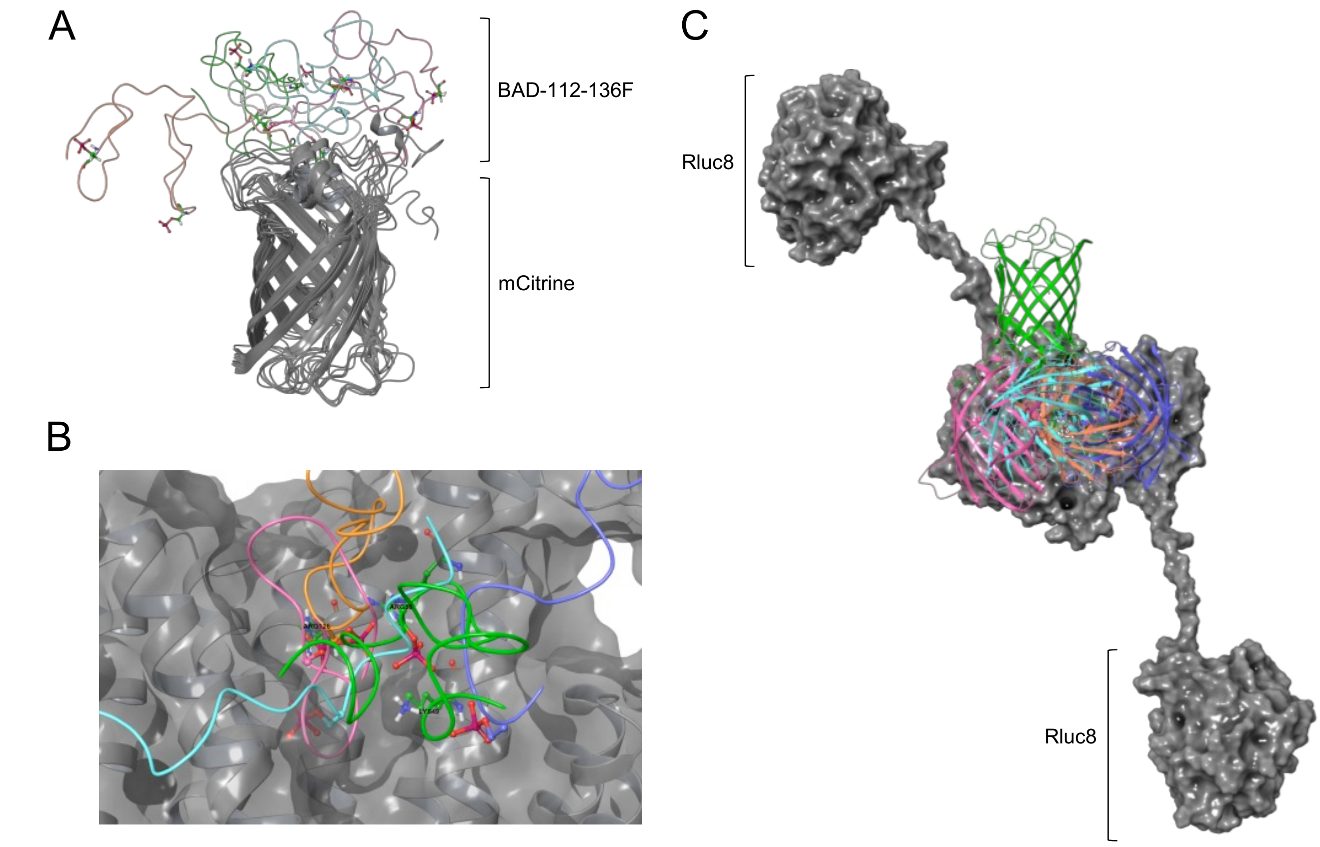

Supplemental Figure 2. Modelling of BAD-112-136F-mCit and docking to 14-3-3ζ-Rluc8.

(**A**) Overlay of top 5 clusters of BAD-112-136F-mCit obtained from GaMD simulations with top cluster in green. (**B**) Zoom in on phosphorylated serine residues of BAD-112-136F-mCit bound to the amphipathic binding groove of 14-3-3ζ-Rluc8 (grey) (**C**) Top 5 clusters of BAD-112-136F-mCit docked to 14-3-3ζ-Rluc8 (grey) with the top pose in green.

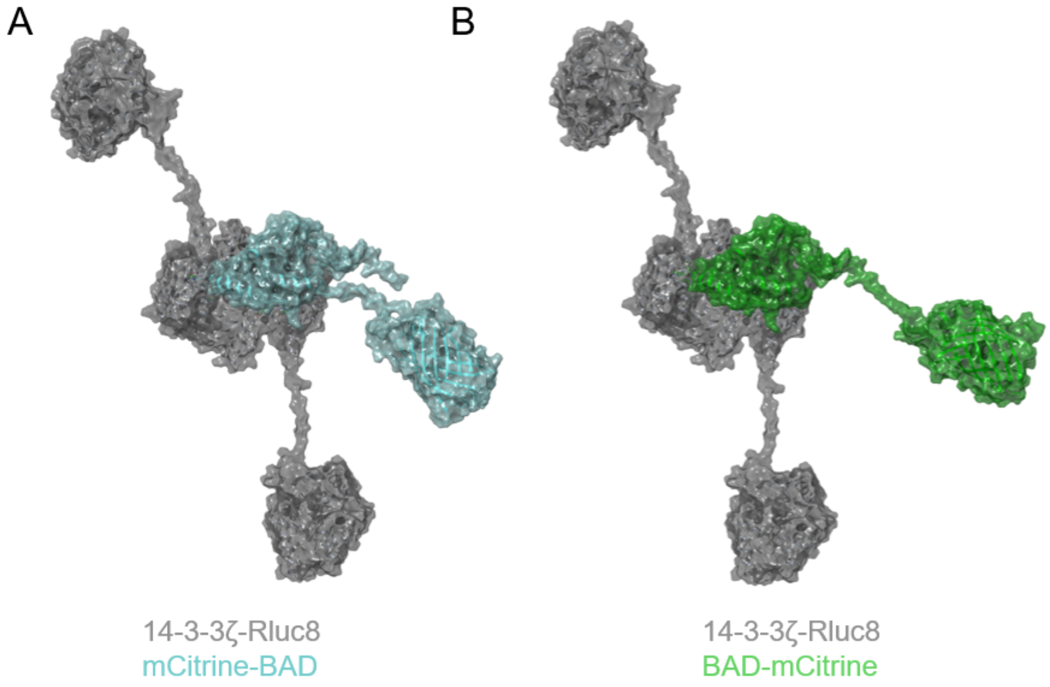

Supplemental Figure 3. Docking of Full length BAD-mCit and mCit-BAD to 14-3-3ζ-Rluc8.

(A) Docked structure of full length BAD (Blue) with mCitrine conjugated to the N-terminus and **(B)** full length bad with mCitrine conjugated to the C-terminus, bound to 14-3-3ζ-Rluc8 (grey)

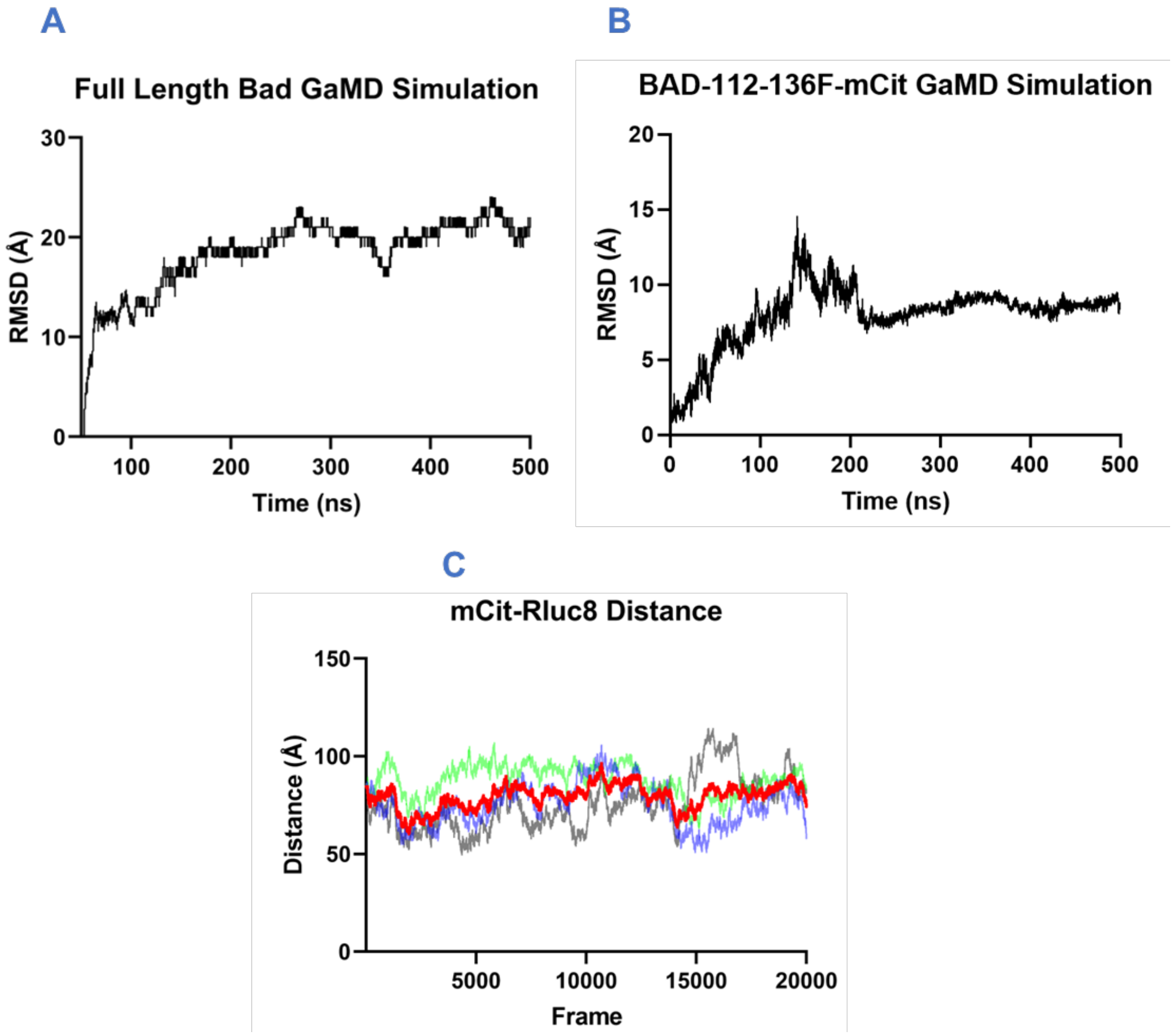

Supplemental Figure 4 Analysis of MD simulations.

(**A**) RMSD (Å) of full length BAD over 500 ns GaMD simulation. (**B**) RMSD of BAD-112-136F-mCit over 500ns GaMD simulation. (**C**) Center of mass distance between Rluc8 and mCitrine on full length BAD-mCit bound to 14-3-3-Rluc8 over 400ns MD simulations Simulation was performed in triplicate with average distance shown in red.

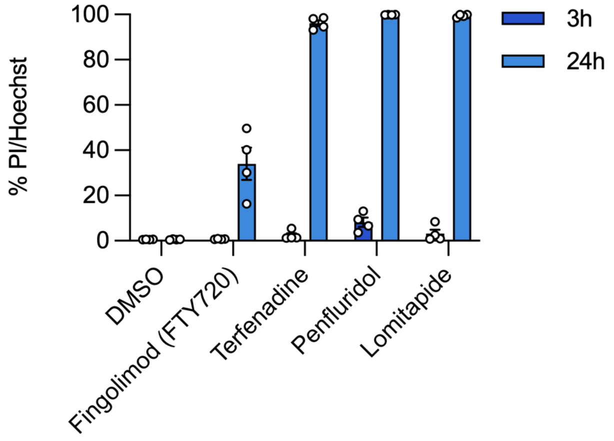

Supplemental Figure 5. The period of time used for BRET measurements is not sufficient to induce cell death.

NIH-3T3 cells were seeded at 80,000 per well in 96-well plates, consistent with the cell density used in BRETscreening assays. At 24 hours post-seeding, compounds were added to the culture media at a final concentration of 20 μM and incubated with cells for 3 hours and 24 hours, respectively. Propidium iodide and hoechst were used to evaluate the cell death.

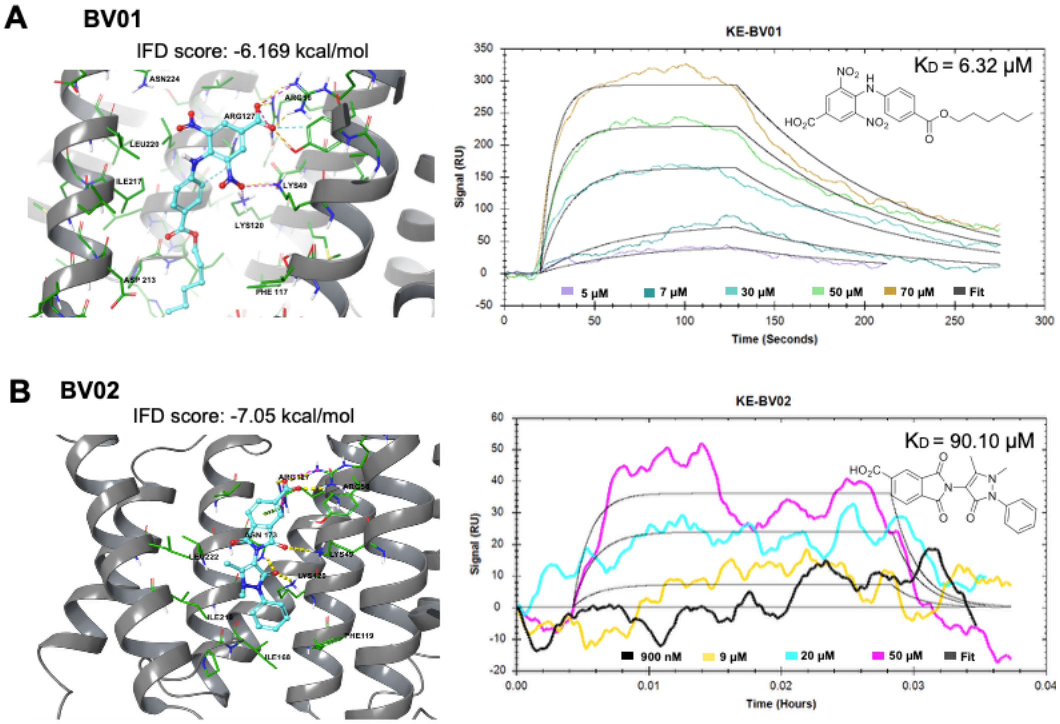

Supplemental Figure 6. Docking and SPR data for BV01 and BV02 suggest specific binding to 14-3-3ζ.

Docking and SPR data for 14-3-3 references BV01 and BV02. Docked structures of drugs are within the amphipathic binding groove of 14-3-3ζ (*PDB*: 2C1J) Residues within 4 Å are shown in green and ligands are shown in cyan. Hydrogen bonds are shown as dashed yellow lines, aromatic H-bonds as blue dashed lines, and π-cation interactions as green dashed lines. SPR assays were conducted by running concentrations between 0.09−50 μM of ligand over immobilized 14-3-3ζ on a high sensitivity carboxylic acid SPR chip (Nicoya); constant or local fitting of the association and dissociation curves were fitted using the software Tracedrawer, black).

IFD score: -7.21 kcal/mol

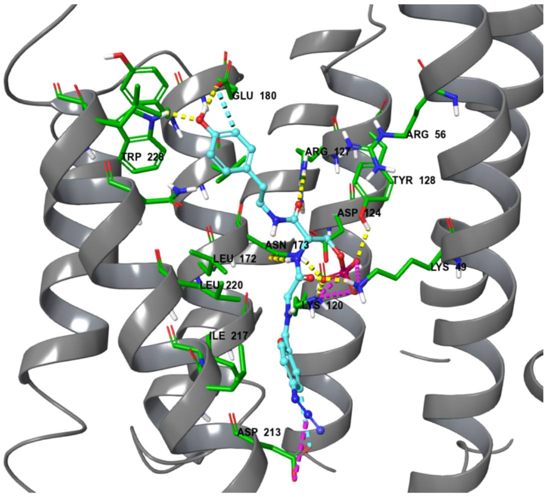

Supplemental Figure 7. Induced fit docking of 1,-2-5 with 14-3-3ζ

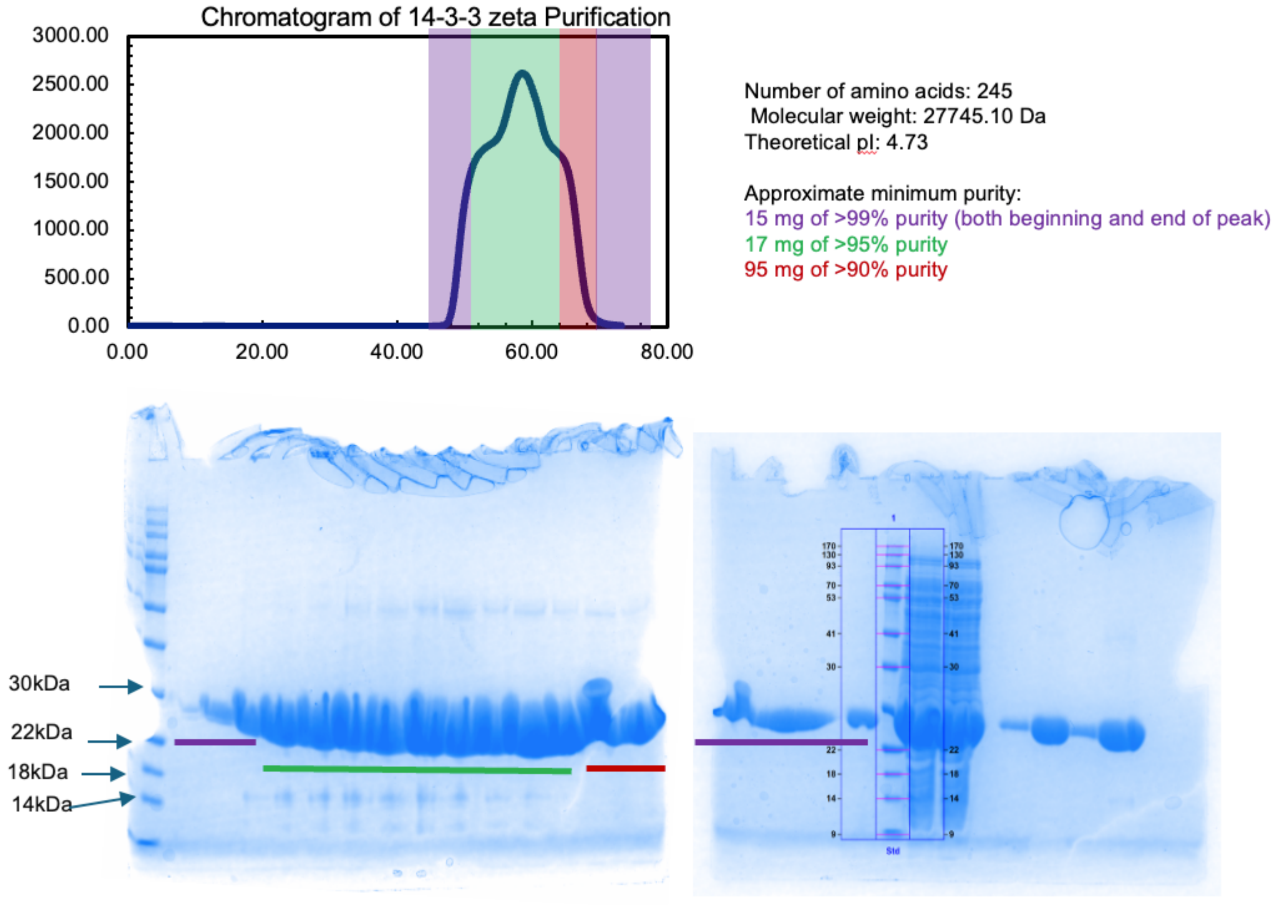

Supplemental Figure 8. Purification of recombinant His-14-3-3*ζ*.

Size exclusion chromatography chromatogram of the protein purification, and Coomassie Blue staining of the fractions obtained. The colours of the lines in the wells indicate which specific fractions were combined into the three batches of protein, note that both the beginning and the end of the peak are combined. The division of the peak by colour in the chromatogram is approximate and only for illustrative purposes. The odd peak shape is likely a factor of the high loading of the column, and the shoulders do not imply significant impurities. The lanes to the right of the ladder are the crude protein pre-purification at different concentrations. Masses of protein were determined by Bradford assay and corrected with the molecular weight.

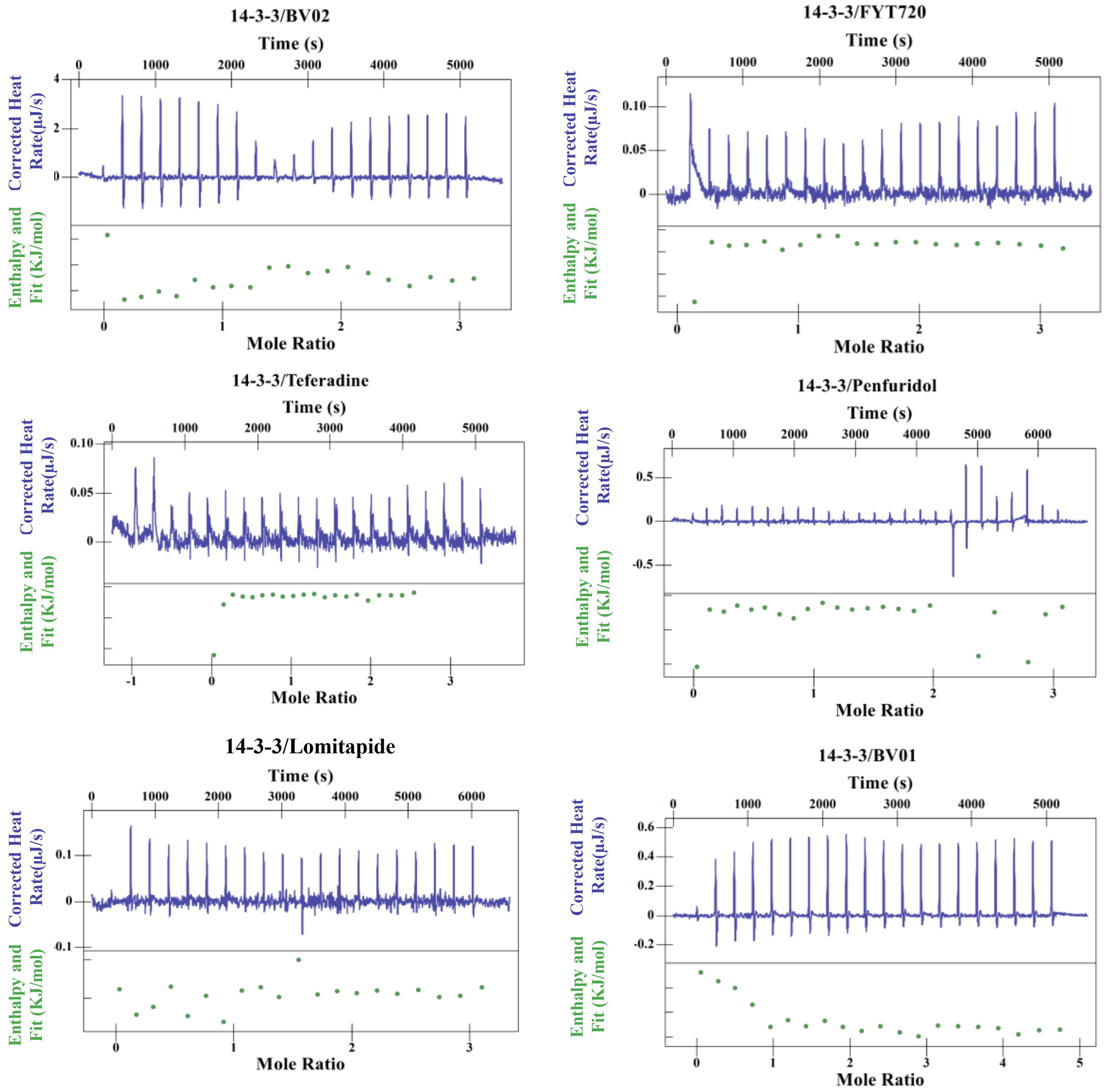

Supplemental Figure 9. Insoluble nature of tested compounds resulted in poor application to isothermal titration calorimetry (ITC) studies.

ITC experiments were performed, but the solubility incompatibility gave unclear data, with a weak sign of binding for BV01 & BV02. All ITC experiments were conducted at 750-1,000 µM of the ligand (in the syringe: *titrant*), with 70 µM of protein in the cell: *titrand*); each injection introduced approximately 4 nmol of ligand. All experiments were conducted at 1-4% DMSO based on drug's solubility.

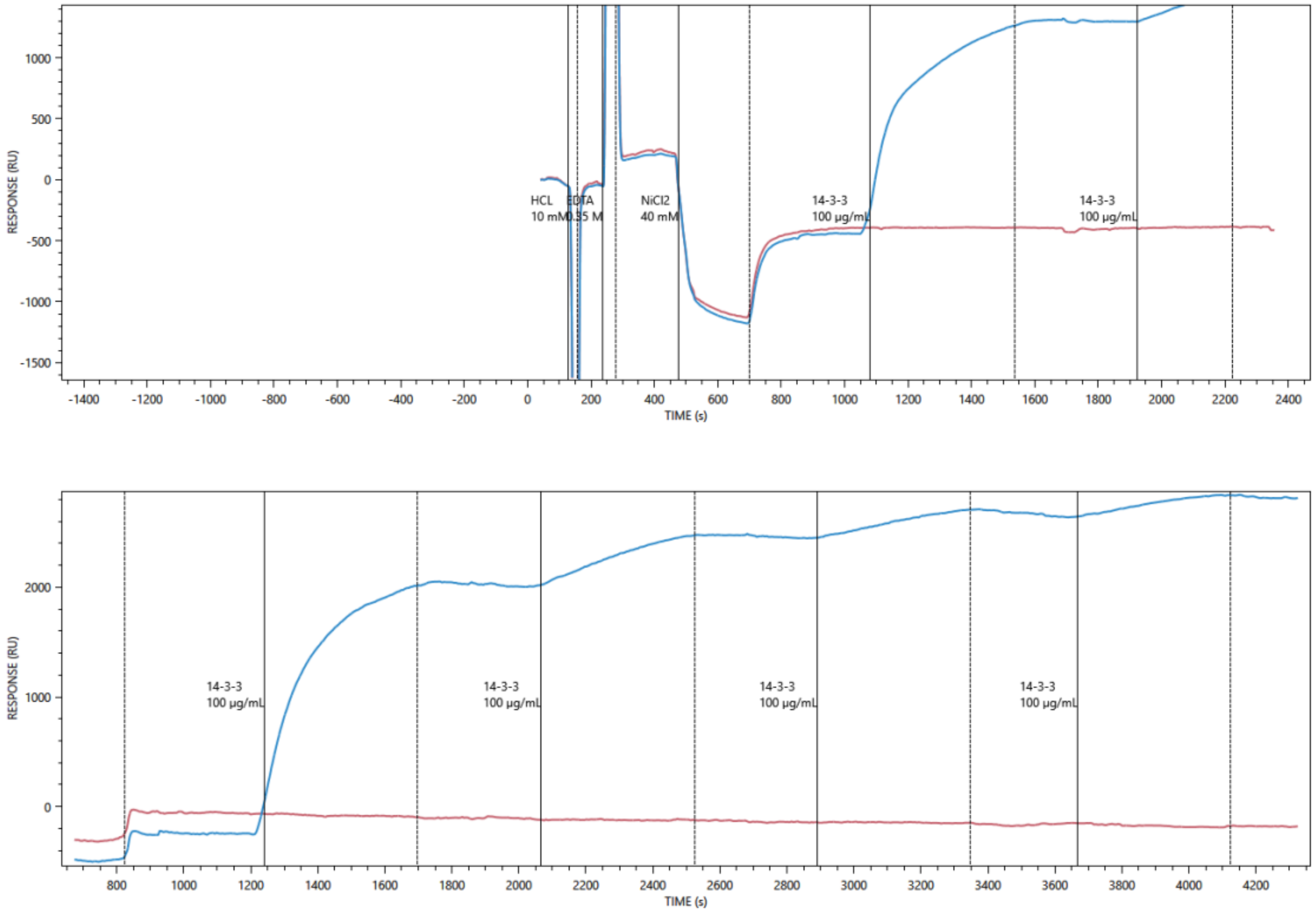

Supplemental Figure 10. HS-NTA sensor activation and protein immobilization for SPR measurements.

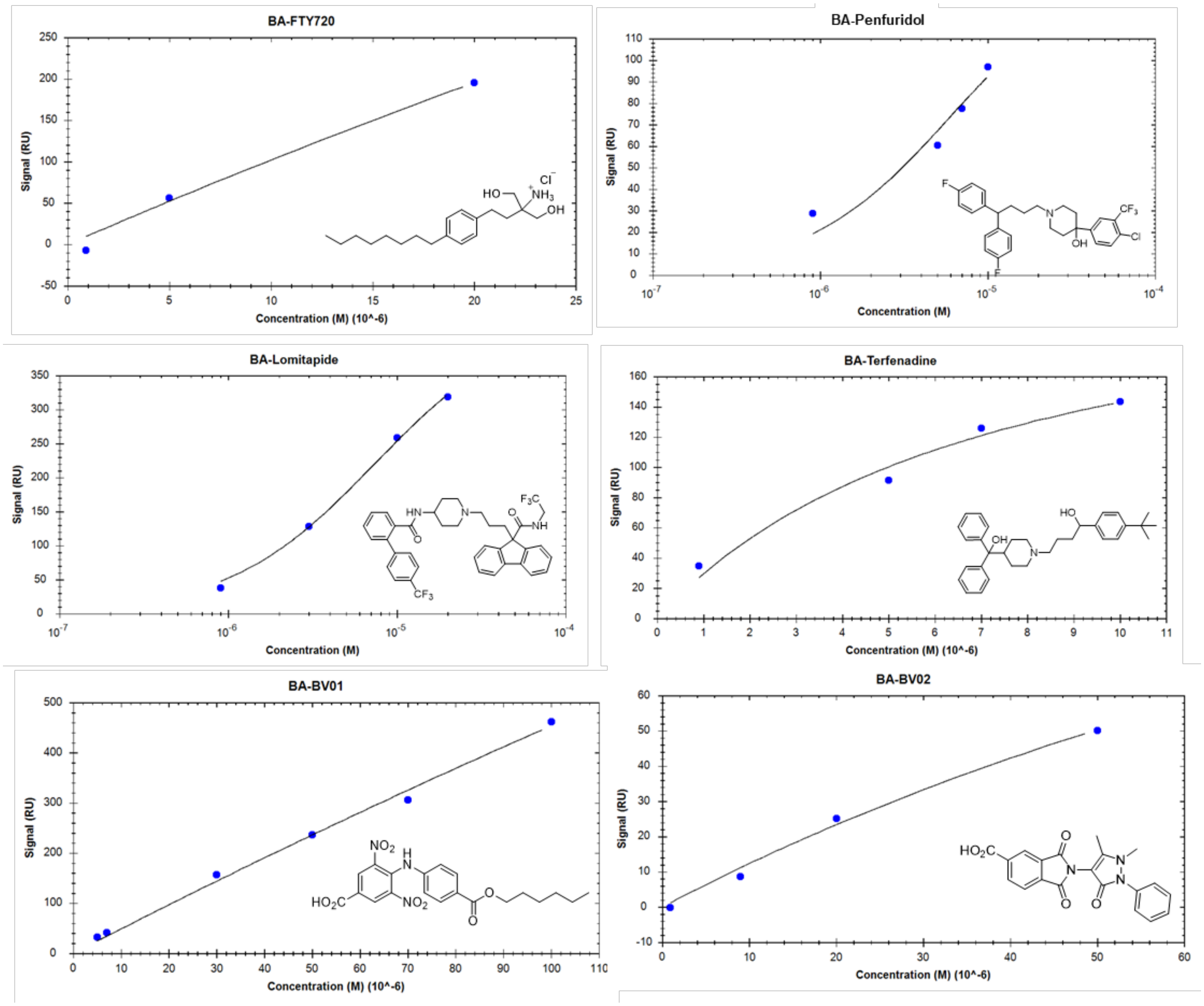

Supplemental Figure 11. Surface plasmon resonance relationship between concentration and signal response of reference compounds and lead candidates.

Derived from the SPR plots for ligand concentrations between 0.09−50 μM running over an immobilized 14-3-3ζ; Langmuir binding isotherm fitted using Tracedrawer.

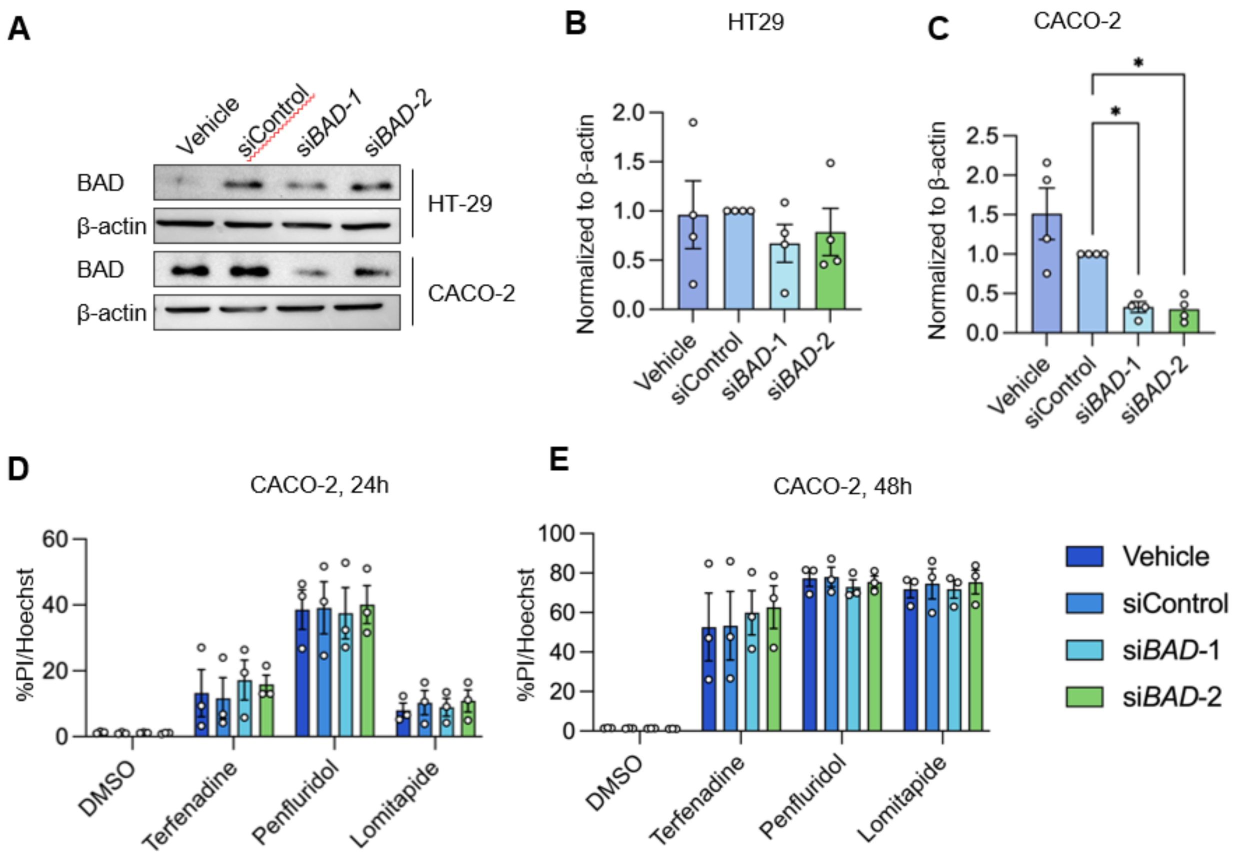

Supplemental Figure 12. Depletion of BAD in CRC cells does not affect the ability of lead candidates to induce cell death.

(A-C) Knockdown efficiency was confirmed by western blotting (A) in HT-29 (B) and CACO-2 (C) cells, followed by densitometric quantification. Protein levels were first normalized to β-actin, and then values were normalized to the siControl group within the same experiment. Representative blots were selected from three independent experiments. Statistical significance was determined using one-way ANOVA with Dunnett's post hoc test. *P < 0.05 compared to siControl. (**D,E**) siRNA-transfected CACO-2 cells were re-plated to 96-well plates and treated with top-hit compounds for 24 hours (D) and 48 hours (E), respectively. Cell death was evaluated using propidium iodide and Hoechst incorporation assay. Data were collected from three independent experiments, each with triplicate measurements. Compared to siControl-transfected cells, no statistically significant differences were observed in panels D and E. Two-way ANOVA was used for statistical analysis.

Synthetic Experimental Details

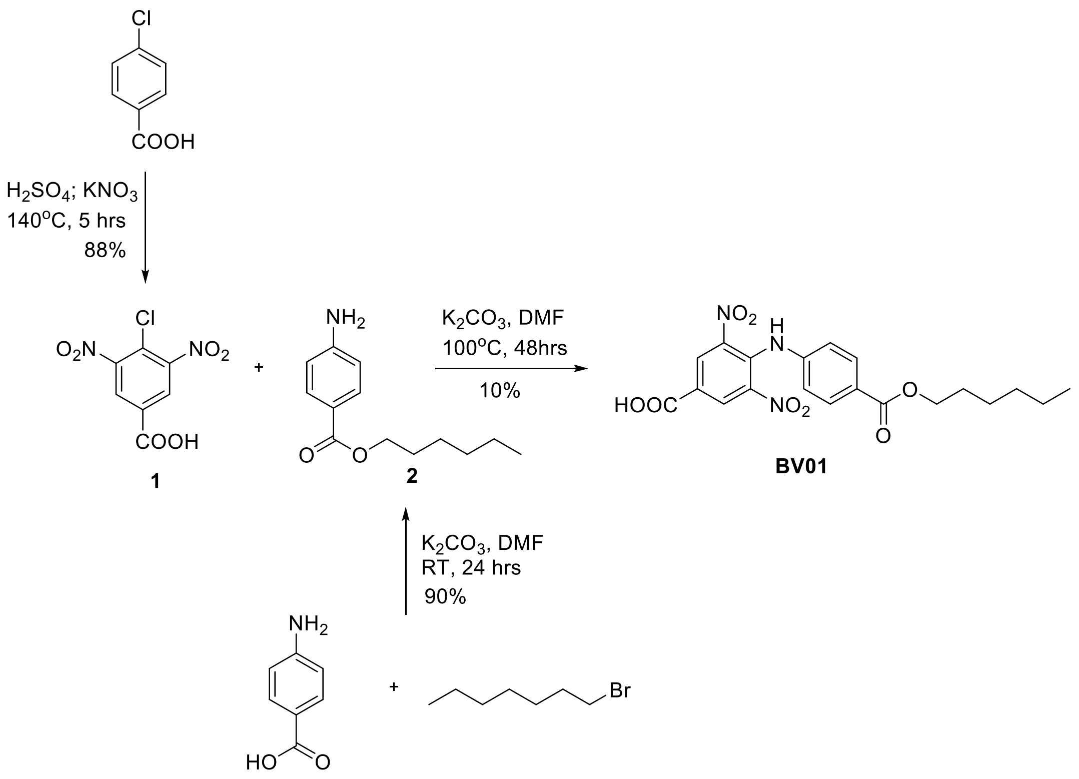

Supplemental Scheme 1. Scheme for the chemical synthesis of BV01

A. Specific protocols for the synthesis of BV01 and starting materials.

A1. Synthesis of 4-chloro-3,5-dinitrobenzoic acid (1)

The compound was prepared according to the protocol of Shang *et al.*[2] with minor modifications. In a round-bottomed flask equipped with a magnetic stirring bar, 4-chlorobenzoic acid (10 g, 60 mmol, 1 equiv.) was dissolved in concentrated H_2SO_4 (42 mL; 0.7 mL/mmol of starting material) under ambient atmosphere. KNO_3 (16.1 g, 150 mmol, 2.5 equiv.) was then added as a solid in portions of ~1g with 5-6 minute intervals, with the temperature maintained under 40 °C at all times using an ambient water bath to assist in dispersing the heat. Once addition was complete, the water bath was replaced with an oil bath and a water-cooled reflux condenser was added, and the mixture was then heated and stirred at 140 °C for 7 hrs; the reaction was monitored by TLC. At 7 hours, when TLC suggested complete conversion, the reaction mixture was cooled to room temperature and then poured into ice-cold water in a 1L beaker, causing white solids to precipitate out. This crude solid was filtered using vacuum

filtration and washed with cold water (4 ºC) multiple times until all suggestions of a green-brown discoloration was gone and only white solid was left. The solids were left overnight in a Buchner funnel under vacuum, and then transferred to a flask and dried on a Schlenk line under high vacuum for 6 hours. This yielded 13.9 g (88%) of **1** as a white solid. It was used in BV01's synthesis without further purification. **TLC:** R_f = 0.65, 4:1 DCM/MeOH; **^{1}H NMR** (500 MHz, DMSO-*d*$_6$) δ = 8.76 (s, 2H). The data is in accordance with the literature.[2, 3]

A2. Synthesis of hexyl 4-aminobenzoate (2)

The compound was prepared according to the protocol of Joshi et al[4] with minor modifications. 4-amino benzoic acid (1 g, 7.29 mmol, 1 equiv.) was dissolved in *N,N*-dimethyl formamide (10 mL) in a round-bottomed flask equipped with a magnetic stirring bar. To this mixture was added 1-bromohexane (1.01 mL. 7.29 mmol, 1 equiv.) followed immediately by solid anhydrous K_2CO_3 (2.5 g, 18.22 mmol, 2.5 equiv.). The flask was sealed with a septum, and the reaction mixture was then stirred for 24hrs at 90 °C under N_2. We monitored the reaction by NMR, and when the methylene protons of the brominated carbon were no longer present, suggesting complete conversion, the reaction was cooled to ambient before being poured into ice-cold water (~30mL) which induced the precipitation of white solids. Methylene chloride (20 mL) was then added, which redissolved the solids, and the phases were separated. The aqueous phase was then re-extracted with an additional 20 mL of methylene chloride; the combined methylene chloride layers were dried with Na_2SO_4 and evaporated *in vacuo* to yield 1.2 g of the title compound as a white solid (90%). This was used in BV01's synthesis without any further purification. **^{1}H NMR** (500 MHz, DMSO-*d*$_6$) δ = 7.62 (d, 2H, *J* = 8.5 Hz), 6.56 (d, 2H, *J* = 8.7 Hz), 5.94 (s, 2H), 4.13 (t, 2H, *J* = 6.6 Hz), 1.61-1.66 (m, 2H), 1.33-1.39 (m, 2H), 1.26-1.30 (m, 4H) and 0.85-0.88 (m, 3H). The data is in accordance with the literature.[4, 5]

A3. Synthesis of 4-((4-((hexyloxy)carbonyl)phenyl)amino)-3,5-dinitrobenzoic acid (BV01)

Compound **1** (100 mg, 0.405 mmol, 1 equiv.) and compound **2** (98.7 mg, 0.455 mmol, 1.1 equiv.) were both dissolved in *N,N*-dimethyl formamide (3 mL) along with anhydrous K_2CO_3 (139.7 mg, 1.012 mmol, 2.5 equiv.). The mixture was then sealed with a septum, and heated to 100 ºC for 48 hrs under N_2. The reaction was monitored *via* NMR, and when conversion didn't proceed further than 30 %, even after adding another equivalency of base, we cooled the reaction and proceeded with work-up. The reaction mixture was quenched with

1M HCl and then extracted with ethyl acetate twice sequentially. The combined organic phase was dried over Na_2SO_4, filtered, and evaporated *in vacuo* to dryness to provide a yellowish-brown crude solid. The crude was then purified by flash chromatography using a mixture of 2.5:2:1 of methylene chloride–hexanes–methanol over silica to yield 17 mg of BV01 as a yellow solid (10%). **TLC:** R_f = 0.8, 2.5:2:1 of DCM/Hex/MeOH; **^{1}H NMR** (500 MHz, CD_3OD-*d_4*) δ = 8.66 (s, 2H), 7.97 (d, 2H, *J* = 8.9 Hz,), 7.81 (d, 2H, *J* = 8.7 Hz), 4.30 (t, 2H, *J* = 6.6 Hz), 1.75-1.80 (m, 2H), 1.44-1.50 (m, 2H), 1.36-1.40 (m, 4H), 0.92-0.94 (m, 3H). The data is in accordance with literature[6].

(Note: We tried to generate BV01 according to the published protocol[6], but were unable to obtain any product or conversion despite increasing the temperature).

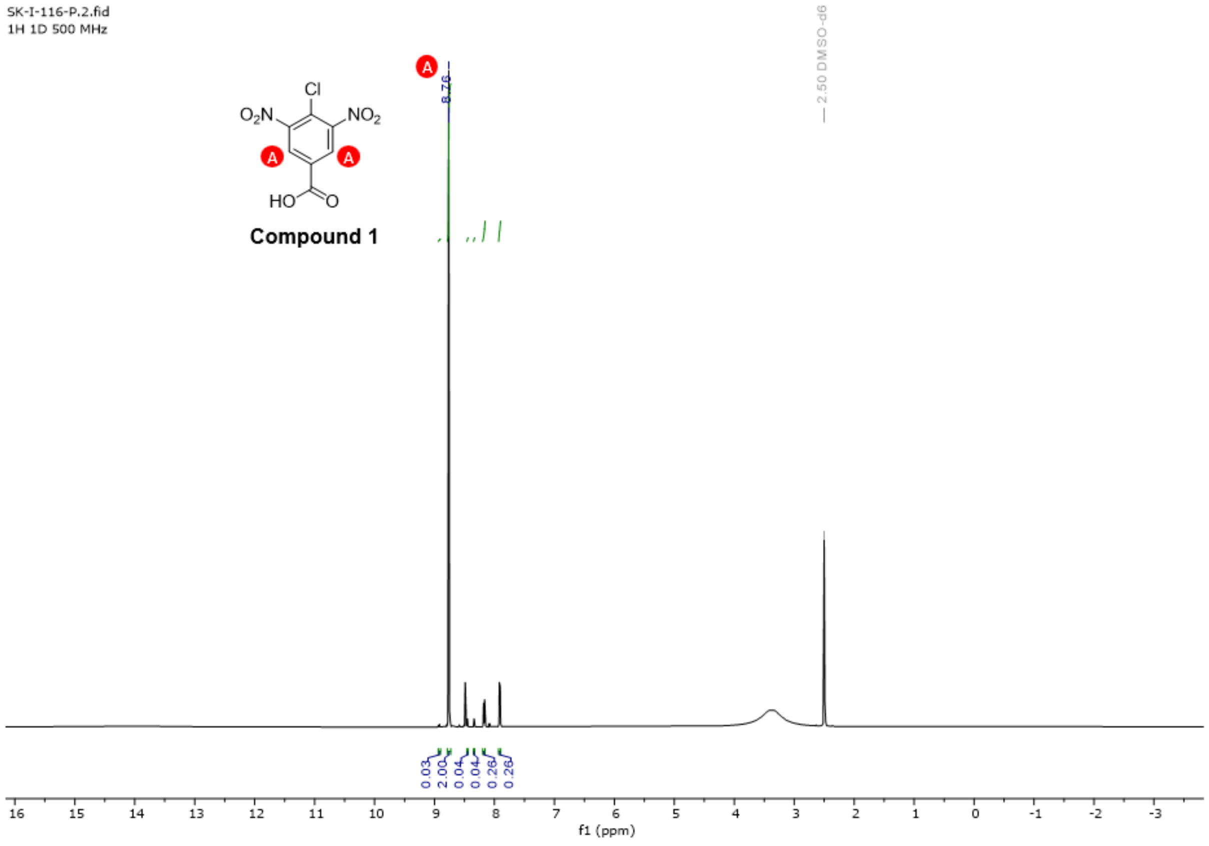

Supplemental Figure 13. ^{1}H NMR spectrum of Compound 1 in DMSO-d_6

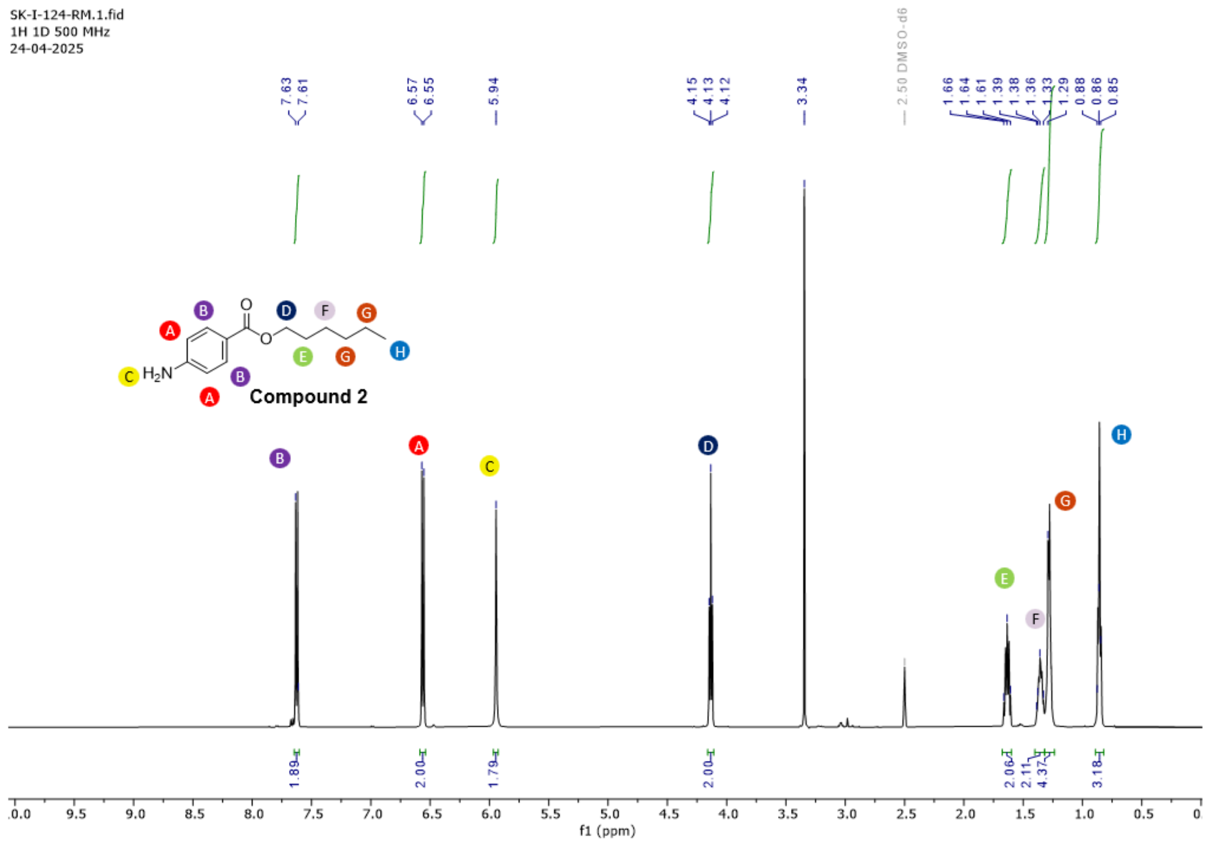

Supplemental Figure 14. **^{1}H NMR spectrum of Compound 2 in DMSO-*d*$_6$**

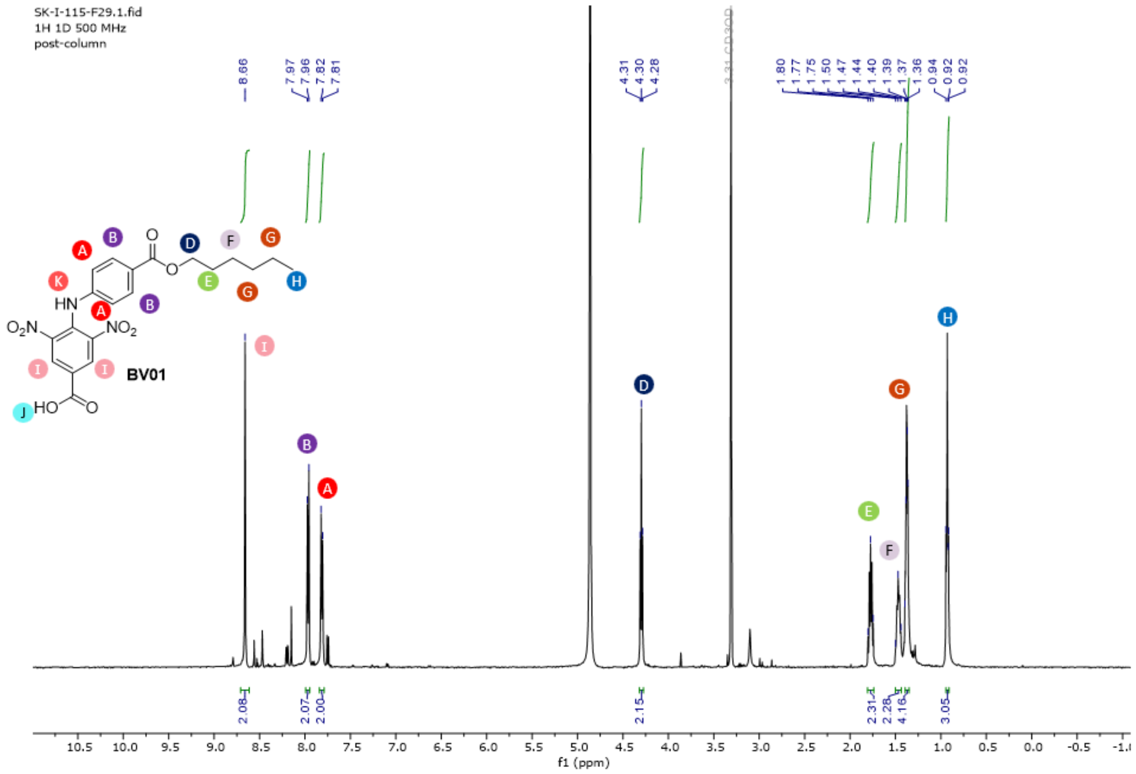

Supplemental Figure 15. ^{1}H NMR spectrum of BV01 in MeOD-d_4

References

1. Iralde-Lorente, L.; Cau, Y.; Clementi, L.; Franci, L.; Tassone, G.; Valensin, D.; Mori, M.; Angelucci, A.; Chiariello, M.; Botta, M., Chemically stable inhibitors of 14-3-3 protein–protein interactions derived from BV02. *Journal of Enzyme Inhibition and Medicinal Chemistry* **2019,** *34* (1), 657-664.
2. Shang, J.; Gallagher, N. M.; Bie, F.; Li, Q.; Che, Y.; Wang, Y.; Jiang, H., aromatic triazole foldamers induced by C–H··· X (X= F, Cl) intramolecular hydrogen bonding. *The Journal of Organic Chemistry* **2014,** *79* (11), 5134-5144.
3. Jia, Y.; Wen, B.; Nan, P.; Chen, H.; Chen, X.; Li, S., Polyphosphoric Acid Catalyzed C− H Dinitration of Passivated Aromatic Compounds with Potassium Nitrate under Mild Condition. *European Journal of Organic Chemistry* **2025,** *28* (1), e202401022.
4. Joshi, V. K.; Joshi, R.; Nakum, K. J.; Katariya, K.; Wani, T.; Hagar, M.; Geres, M.; Bhatt, R., Self-assemblies of two ring salicylaldimine at low temperatures: Mesophase behaviour and DFT investigations. *Journal of Molecular Structure* **2025,** *1333*, 141742.
5. Mori, M.; Vignaroli, G.; Cau, Y.; Dinić, J.; Hill, R.; Rossi, M.; Colecchia, D.; Pešić, M.; Link, W.; Chiariello, M., Discovery of 14-3-3 protein–protein interaction inhibitors that sensitize multidrug-resistant cancer cells to doxorubicin and the Akt inhibitor GSK690693. *ChemMedChem* **2014,** *9* (5), 973-983.
6. Corradi, V.; Mancini, M.; Santucci, M. A.; Carlomagno, T.; Sanfelice, D.; Mori, M.; Vignaroli, G.; Falchi, F.; Manetti, F.; Radi, M., Computational techniques are valuable tools for the discovery of protein–protein interaction inhibitors: The 14-3-3σ case. *Bioorganic & medicinal chemistry letters* **2011,** *21* (22), 6867-6871.
